# Enhanced Multifunctional Properties of Bipyridyl-Containing Polyurethane Nanocomposites Reinforced with Graphene/ZnO Hybrid Fillers

**DOI:** 10.3390/polym18182191

**Published:** 2026-09-08

**Authors:** Jing-Lun Chen, Yun-Shao Huang, Wen-Chin Tsen, Chi-Hui Tsou, Chin-Wen Chen, Maw-Cherng Suen

**Affiliations:** 1 Graduate School of Fabric Technology Management, Lee-Ming Institute of Technology, Taipei 243083, Taiwan; lit10641@mail.lit.edu.tw (J.-L.C.); charity@mail.lit.edu.tw (W.-C.T.); 2 Department of Applied Cosmetology, Lee-Ming Institute of Technology, Taipei 243083, Taiwan; lit00627@mail.lit.edu.tw; 3 School of Materials Science and Engineering, Sichuan University of Science and Engineering, Zigong 643000, China; mayko0301@hotmail.com; 4 Department of Molecular Science and Engineering, Institute of Organic and Polymeric Materials, Research and Development Center of Smart Textile Technology, National Taipei University of Technology, Taipei 10608, Taiwan; cwchen@ntut.edu.tw

**Keywords:** polyurethane nanocomposites, bipyridine chain extender, graphene/zinc oxide hybrid filler, X-ray photoelectron spectroscopy, surface wettability, antibacterial activity

## Abstract

A series of polyurethane (PU) nanocomposites incorporating 4,4′-bis(hydroxymethyl)-2,2′-bipyridine (BBD) as a chain extender and a commercially supplied graphene/zinc oxide (G/ZnO) hybrid filler were successfully synthesized. The effects of G/ZnO loading (0–2.0 wt.%) on the structural, thermal, mechanical, surface-wettability, and antibacterial properties of the nanocomposites were systematically investigated. Fourier-transform infrared spectroscopy confirmed the formation of the polyurethane structure and revealed changes in characteristic absorption bands following G/ZnO incorporation. Morphological observation of the pristine G/ZnO hybrid filler revealed an irregular and aggregated morphology, while X-ray diffraction confirmed the presence of crystalline ZnO. Energy-dispersive X-ray spectroscopy and elemental mapping showed Zn-containing regions within the examined areas of the G/ZnO-containing PU samples. X-ray photoelectron spectroscopy further confirmed the surface presence of Zn-containing species, with the Zn atomic concentration increasing from 0 at.% in PU-01 to 0.82 at.% in PU-04. Thermogravimetric analysis showed modest changes in thermal decomposition behavior with increasing G/ZnO loading, while differential scanning calorimetry and dynamic mechanical analysis revealed shifts in glass-transition and relaxation behavior, consistent with changes in polymer-chain mobility and the local interfacial environment. The tensile strength increased from 2.68 MPa for neat PU to 11.76 MPa for the nanocomposite containing 2.0 wt.% G/ZnO, accompanied by an increase in Young’s modulus. The water contact angle increased from approximately 68° to 89°, indicating reduced apparent surface wettability with increasing G/ZnO loading. The nanocomposites also exhibited antibacterial activity against Escherichia coli and Staphylococcus aureus, with antibacterial efficiencies exceeding 95% at higher G/ZnO loadings. Overall, the incorporation of the commercial G/ZnO hybrid filler was associated with changes in the thermal, mechanical, surface, and antibacterial properties of the BBD-containing PU system. Because separate PU systems without BBD and individual graphene- and ZnO-containing controls were not included, the individual contributions of BBD, graphene, and ZnO, as well as any synergistic effect between graphene and ZnO, cannot be established from the present results. Further studies addressing filler leaching, long-term antibacterial stability, coating adhesion, environmental durability, and cytocompatibility are required to establish the practical applicability of these materials.

## 1. Introduction

Polyurethane (PU) is one of the most versatile engineering polymers owing to its segmented molecular architecture, excellent elasticity, abrasion resistance, chemical resistance, adhesion, and processability [1,2,3,4]. These characteristics have enabled its widespread use in coatings, elastomers, adhesives, membranes, biomedical devices, smart textiles, wearable electronics, and flexible sensors [2,3,4,5,6]. By adjusting the chemical composition and relative proportions of the soft and hard segments, PU can be tailored to exhibit a wide range of mechanical, thermal, surface, and functional properties. However, emerging applications increasingly require PU materials to combine mechanical strength, thermal stability, environmental durability, and biological functionality without compromising processability or structural integrity. Accordingly, molecular design and nanocomposite approaches have been widely explored to integrate structural reinforcement, antimicrobial activity, environmental resistance, and stimuli-responsive behavior within a single PU system [3,4,5,7,8,9].

Among various nanofillers, graphene has attracted considerable attention because of its exceptional mechanical and thermal properties [10,11,12,13]. Its incorporation into PU matrices can improve load transfer, constrain segmental motion, and influence microphase organization, thereby enhancing mechanical and thermomechanical performance [14,15,16,17,18,19]. Nevertheless, strong van der Waals attraction and π–π stacking between graphene sheets often cause aggregation and reduce the accessible interfacial area [11,12,15,16,17,18,19]. Achieving stable graphene distribution and effective adhesion to the polymer matrix therefore remains an important challenge.

ZnO nanoparticles are also widely used as multifunctional inorganic fillers because of their antibacterial activity, ultraviolet-shielding capability, thermal stability, and compatibility with polymer matrices [7,20,21,22,23]. In PU nanocomposites, ZnO can improve antibacterial performance, stiffness, thermal stability, and surface characteristics [7,21,22,23]. However, its high surface energy frequently promotes particle agglomeration, which decreases the effective surface area and limits interaction with the surrounding polymer chains [21,22,23,24,25]. Appropriate control of the ZnO–polymer interface is therefore required to fully exploit its multifunctional potential [21,22,23,24,25,26].

Graphene/ZnO (G/ZnO) hybrid nanostructures provide a promising route to address the limitations of the individual components. The deposition or association of ZnO with graphene can suppress graphene restacking, while the graphene substrate can reduce ZnO agglomeration. As a result, G/ZnO hybrids may exhibit improved distribution, interfacial compatibility, and multifunctional performance compared with the separate fillers [27,28,29]. However, these advantages depend strongly on the interactions established between the hybrid nanofillers and the PU matrix [15,21,27,28,29].

The performance of PU nanocomposites is largely determined by the quality of the polymer–nanofiller interface. Effective interfaces promote stress transfer, reduce filler aggregation, and alter the local organization of polymer segments [10,11,12,14,15,16,17,18,19,21]. In segmented PU systems, hydrogen bonding and dipole-related interactions contribute to hard-segment cohesion and microphase organization [4,30,31,32]. In graphene-containing composites, aromatic groups may adsorb onto graphene surfaces through π–π interactions [10,11,12,15], whereas pyridyl groups may provide interaction or coordination sites for metal-containing domains [31,33]. Introducing functional molecular units directly into the PU backbone may therefore provide more effective interfacial control than conventional physical blending alone.

Pyridyl-containing chain extenders are particularly attractive because pyridine nitrogen atoms can participate in hydrogen bonding, dipole–dipole interactions, and reversible coordination with metal-containing species [31,33]. Among them, 4,4′-bis(hydroxymethyl)-2,2′-bipyridine (BBD) possesses a bifunctional structure suitable for PU synthesis. Its hydroxymethyl groups react with isocyanate groups and become incorporated into the hard segments, while the rigid bipyridyl framework introduces nitrogen-containing sites that may interact with Zn-containing nanostructures. Compared with conventional aliphatic chain extenders, BBD may also enhance hard-segment organization through its rigid aromatic structure [31]. In addition, its pyridine nitrogen atoms may serve as potential coordination sites for Zn-containing domains, consistent with previously reported coordination-assisted PU systems [33]. The aromatic bipyridyl framework may also interact with graphene surfaces through π–π interactions, although this remains a proposed mechanism in the present material system [10,11,12,15].

Although graphene-reinforced PU, ZnO-containing PU, and G/ZnO hybrid nanocomposites have been extensively investigated, the incorporation of G/ZnO hybrids into BBD-containing PU has received limited attention [7,14,15,16,17,18,19,21,22,23,24,25,26,27,28,29]. In particular, the combined influence of hydrogen bonding, aromatic interactions, dipole-related interactions, and possible Zn–pyridyl coordination on the thermal, mechanical, surface, and antibacterial properties of these materials has not been systematically clarified. Most previous studies have focused on individual functions, such as mechanical reinforcement, thermal conductivity, photocatalytic activity, or antibacterial behavior, rather than examining how a functional chain extender and hybrid nanofiller jointly affect multiple properties. Therefore, the development of a BBD-containing PU/G/ZnO system provides an opportunity to investigate whether complementary molecular interactions can improve overall nanocomposite performance.

In this study, multifunctional PU nanocomposites were synthesized through a conventional two-step polymerization process using BBD as a chain extender and a commercially supplied G/ZnO hybrid filler. The effects of G/ZnO loading on chemical structure, filler distribution, thermal degradation behavior, thermomechanical behavior, mechanical properties, surface wettability, and antibacterial performance were systematically evaluated. Particular attention was given to composition-dependent changes associated with increasing G/ZnO hybrid-filler loading. Possible intermolecular and interfacial interactions are discussed as potential contributing factors rather than as experimentally established mechanisms. The present study therefore provides fundamental information on the structure–property relationships of G/ZnO-containing polyurethane nanocomposites under the investigated laboratory conditions.

## 2. Experimental Section

### 2.1. Materials

4,4′-Diphenylmethane diisocyanate (MDI) and polycaprolactone diol (PCL) were purchased from Sigma-Aldrich (St. Louis, MO, USA). 4,4′-Bis(hydroxymethyl)-2,2′-bipyridine (BBD) was purchased from Tokyo Chemical Industry (TCI, Tokyo, Japan). *N*,*N*-Dimethylacetamide (DMAc) was purchased from Mallinckrodt Chemicals (Dublin, Ireland). Graphene/zinc oxide hybrid nanofillers (G/ZnO) were supplied by Apex Nano Co., Ltd. (New Taipei City, Taiwan). According to the supplier-provided specification, the commercially supplied G/ZnO hybrid filler contained approximately 33 wt.% carbon component and 67 wt.% ZnO, corresponding to a nominal carbon-to-ZnO mass ratio of approximately 1:2. This composition represents the formulation of the commercial product and was not independently optimized in the present study. All chemicals were used as received without further purification.

### 2.2. Synthesis of BBD/G/ZnO/PU Nanocomposites

**Scheme 1:**
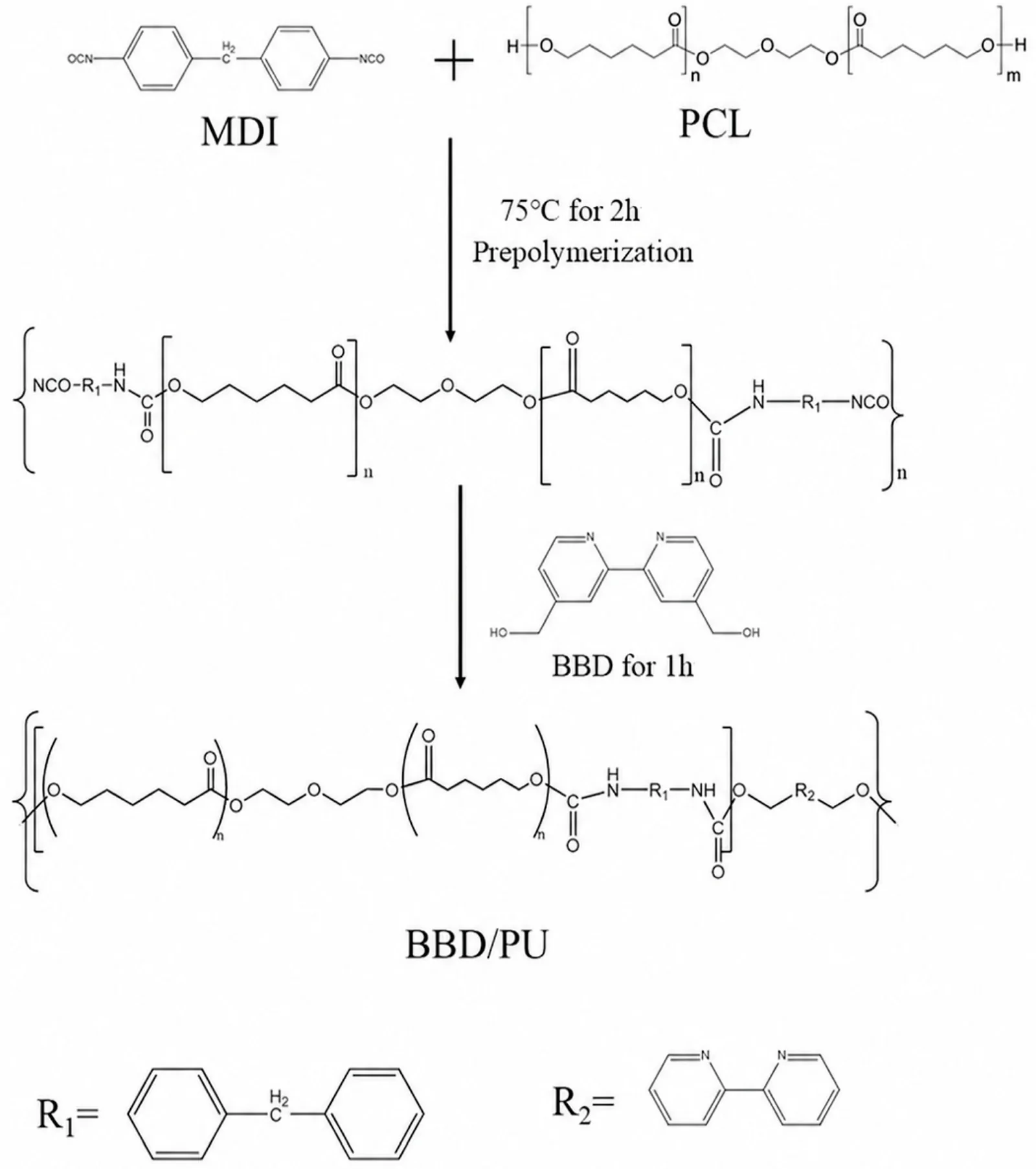
Schematic illustration of the synthesis and chemical structure of polyurethane (PU).

The synthesis procedure for the BBD/G/ZnO/PU nanocomposites is schematically illustrated in Scheme 1 and was carried out via a conventional two-step solution polymerization method [1,4,31,32]. First, polycaprolactone diol (PCL, number-average molecular weight *M*_n_ = 530 g·mol^−1^) and 4,4′-diphenylmethane diisocyanate (MDI) were introduced into a four-neck flask at a predetermined molar ratio. Using anhydrous *N*,*N*-dimethylacetamide (DMAc) as the reaction solvent, the mixture was reacted at 75 °C under continuous stirring at 300 rpm for 2 h under a nitrogen atmosphere to produce an isocyanate (–NCO)-terminated prepolymer [1,4,31]. Subsequently, 4,4′-bis(hydroxymethyl)-2,2′-bipyridine (BBD) was introduced as the chain extender, and the polymerization was continued for an additional 1 h at the same temperature to obtain the bipyridyl-containing polyurethane (PU) matrix [31,33].

In this synthesis formulation, the molar ratio of MDI:PCL:BBD was fixed at 4:3:1. This composition was selected based on previous studies demonstrating that appropriate regulation of the hard-segment content effectively balances mechanical strength, thermal stability, and chain mobility in segmented polyurethane systems [4,31,32,33,34]. The synthetic route is illustrated in Scheme 1, and the corresponding hard- and soft-segment contents were approximately 43.36 wt.% and 56.64 wt.%, respectively, as summarized in Table 1.

For the fabrication of the nanocomposites, the commercially supplied graphene/zinc oxide (G/ZnO) hybrid powder with a nominal graphene/carbon-to-ZnO mass ratio of approximately 1:2 was first dispersed in DMAc and ultrasonicated for 15 min. The resulting suspension was then added to the freshly prepared PU solution and stirred at room temperature for 10 min. The composite solution was subsequently cast onto a flat Teflon mold and degassed before being dried in a forced-air oven at 70 °C for 48 h to remove the residual solvent. After peeling from the mold, the BBD/G/ZnO/PU nanocomposite films were obtained. G/ZnO nanohybrids were incorporated into the PU matrix at loadings of 0, 0.5, 1.0, and 2.0 wt.%, respectively (Table 2), to systematically investigate the influence of nanofiller content on the structure, interfacial interactions, and multifunctional properties of the resulting nanocomposites [15,16,17,18,19,21,22,23,24,25,26,27,28,29].

### 2.3. Gel Permeation Chromatography (GPC)

The molecular-weight distribution of the synthesized polyurethane matrix was determined by gel permeation chromatography (GPC) using a Waters 1515 high-performance liquid chromatography system equipped with a Waters 2414 refractive-index detector (Waters Corporation, Milford, MA, USA), following procedures commonly adopted for polyurethane characterization [22,23]. Approximately 0.01 g of the BBD/G/ZnO/PU-01 sample was dissolved in HPLC-grade *N*-methyl-2-pyrrolidone (NMP) under continuous stirring for at least 6 h to ensure complete dissolution. Before injection, the solution was filtered through a solvent-resistant membrane filter to remove undissolved particles. GPC measurements were performed at 40 °C using NMP as the mobile phase. The number-average molecular weight (Mn), weight-average molecular weight (Mw), and dispersity (PDI = Mw/Mn) were calculated using the instrument calibration software (Waters Corporation, Milford, MA, USA).

### 2.4. Fourier Transform Infrared Spectroscopy (FTIR) Characterization

Fourier transform infrared (FTIR) spectroscopy was performed to characterize the chemical structures and intermolecular interactions of the polyurethane nanocomposites [4,15,31]. FTIR spectra were recorded using a Spotlight 200i Sp2 spectrometer equipped with an AutoATR system (PerkinElmer, Waltham, MA, USA). The spectra were collected over the wavenumber range of 4000–650 cm^−1^ at a resolution of 2 cm^−1^, and each spectrum represented the average of 16 scans.

### 2.5. Energy-Dispersive X-Ray Spectroscopy (EDS)

Energy-dispersive X-ray spectroscopy (EDS) was employed to determine the elemental composition and spatial distribution of the nanofillers within the polyurethane matrix [21,29]. The surface elemental composition of the PU films (1 × 1 cm^2^) was examined using a TM4000 Plus scanning electron microscope (SEM; Hitachi High-Tech Corporation, Tokyo, Japan) equipped with an X-stream-2 EDS system (Oxford Instruments, Abingdon, UK). EDS elemental mapping and point analyses were performed simultaneously during morphological observations. Qualitative and quantitative EDS analyses were conducted using AZtec software (Oxford Instruments, Abingdon, UK).

### 2.6. X-Ray Diffraction (XRD) Characterization

X-ray diffraction (XRD) analysis was carried out to examine the crystalline structures of the synthesized nanocomposites and to identify the crystalline phases of ZnO within the polyurethane matrix [7,21,28]. The diffraction patterns were recorded using a D8 ADVANCE ECO X-ray diffractometer (Bruker AXS GmbH, Karlsruhe, Germany) with Cu Kα radiation (λ = 1.5406 Å) over a 2θ range of 10–70°. The diffraction peaks were indexed according to Bragg’s law to evaluate the crystallinity and crystal structures of the synthesized materials.

### 2.7. X-Ray Photoelectron Spectroscopy (XPS)

X-ray photoelectron spectroscopy (XPS) was performed using a ULVAC-PHI Quantera spectrometer (ULVAC-PHI, Inc., Chigasaki, Kanagawa, Japan) equipped with a monochromatic Al Kα X-ray source (1486.6 eV). Survey spectra were recorded over a binding-energy range of 0–1400 eV. The binding energies were calibrated using the C 1s peak at 284.8 eV as the internal reference [35]. High-resolution C 1s, O 1s, N 1s, and Zn 2p spectra were acquired to investigate the surface elemental composition and chemical states of the constituent elements.

### 2.8. Thermogravimetric Analysis (TGA) Characterization

Thermogravimetric analysis (TGA) was performed to evaluate the thermal stability and decomposition behavior of the synthesized polyurethane nanocomposites following commonly reported procedures for polyurethane-based composites [4,7,15,19]. The measurements were carried out using an STA 7200 thermal analyzer (Hitachi, Tokyo, Japan). Approximately 3.0–8.0 mg of each sample was placed in a ceramic crucible and heated from 25 to 700 °C at a constant heating rate of 10 °C min^−1^ under a continuous nitrogen atmosphere. The sample weight loss was continuously recorded as a function of temperature. TGA measurements were performed once for each sample; therefore, statistical uncertainties or standard deviations are not reported for the thermal degradation parameters.

### 2.9. Differential Scanning Calorimetry (DSC)

Differential scanning calorimetry (DSC) was employed to investigate the thermal transitions and segmental mobility of the polyurethane nanocomposites [4,15,30,31]. Measurements were conducted using a DSC 7000X instrument (Hitachi, Tokyo, Japan). Approximately 3.0–8.0 mg of each sample was sealed in an aluminum pan. The specimens were heated and cooled between −100 and 200 °C at a rate of 10 °C min^−1^. To eliminate the previous thermal history, the samples were first heated above their melting temperature and maintained isothermally for 5 min before the subsequent cooling and second heating cycles.

### 2.10. Dynamic Mechanical Analysis (DMA) Characterization

Dynamic mechanical analysis (DMA) was carried out to evaluate the viscoelastic behavior and glass transition characteristics of the polyurethane nanocomposites [4,15,30,32]. The measurements were performed using a DMS 6100 dynamic mechanical analyzer (Tech Max, Tokyo, Japan). Rectangular specimens (20 × 5 × 0.2 mm^3^) were tested in tensile mode at a frequency of 1 Hz with an oscillation amplitude of 5 μm. The temperature was increased from −50 to 50 °C at a heating rate of 3 °C min^−1^. The storage modulus (E′), loss modulus (E″), and damping factor (tan δ) were continuously recorded to determine the glass transition temperature (Tg).

### 2.11. Tensile Testing

The mechanical properties of the polyurethane nanocomposites were evaluated by tensile testing in accordance with ASTM D882-18 [36]. and previously reported procedures for polyurethane materials [15,16,18,32]. Tensile tests were performed using a CY-6040A4 universal testing machine (Jun Yan Precision Machinery Co., Ltd., Taichung, Taiwan). Rectangular film specimens (45 × 8 × 0.2 mm^3^) were tested at ambient temperature with a crosshead speed of 50 mm min^−1^. The tensile strength, Young’s modulus, and elongation at break were calculated from the stress–strain curves. Mechanical testing was performed using three specimens for each composition (*n* = 3), and the results are expressed as the mean ± standard deviation (SD). No inferential statistical significance testing was performed.

### 2.12. Water Contact Angle (WCA) Measurement

Static water contact angle (WCA) measurements were performed to evaluate the surface wettability of the polyurethane nanocomposites following commonly reported procedures [15,21,37]. Measurements were conducted using a HO-IAD-CAM-01A contact angle goniometer (Tianxiang Technology Instrument Co., Ltd., Taipei, Taiwan) at room temperature. A droplet of deionized water (approximately 2–5 μL) was gently deposited on the film surface, and the contact angle was recorded using an integrated image acquisition system. The reported value for each sample represents the average of at least three to four independent measurements obtained at different surface locations.

### 2.13. Antibacterial Test

The antibacterial activities of the synthesized polyurethane nanocomposites were evaluated against the Gram-negative bacterium *Escherichia coli* and the Gram-positive bacterium *Staphylococcus aureus* using the colony-forming unit (CFU) counting method according to procedures widely adopted for ZnO- and graphene-based antibacterial polymer nanocomposites [23,24,29,38,39,40,41,42,43]. Briefly, 1.0 g of each neat PU or BBD/G/ZnO/PU nanocomposite sample was immersed in 25 mL of nutrient broth inoculated with either *E. coli* or *S. aureus* (1 × 10^5^ CFU mL^−1^) and incubated at 37 °C for 24 h under static conditions. Subsequently, a 5 μL aliquot of the bacterial suspension was serially diluted tenfold and spread onto nutrient agar plates. After further incubation at 37 °C for 24 h, the number of viable bacterial colonies was counted to determine the colony-forming units (CFU). The antibacterial efficiency was calculated using Equation (1):

(1) $$Antibacterial\;efficiency\;(\%)=\frac{A-B}{A}\;\times \;100\%$$

where *A* and *B* represent the viable bacterial counts (CFU) of the neat PU and BBD/G/ZnO/PU nanocomposite samples, respectively. All antibacterial experiments were conducted independently for *E. coli* and *S. aureus* under identical experimental conditions. Each antibacterial measurement was performed in triplicate (*n* = 3), and the quantitative results are presented as the mean ± standard deviation (SD).

## 3. Results and Discussion

### 3.1. Characterization of the G/ZnO Hybrid Filler

**Figure 1:**
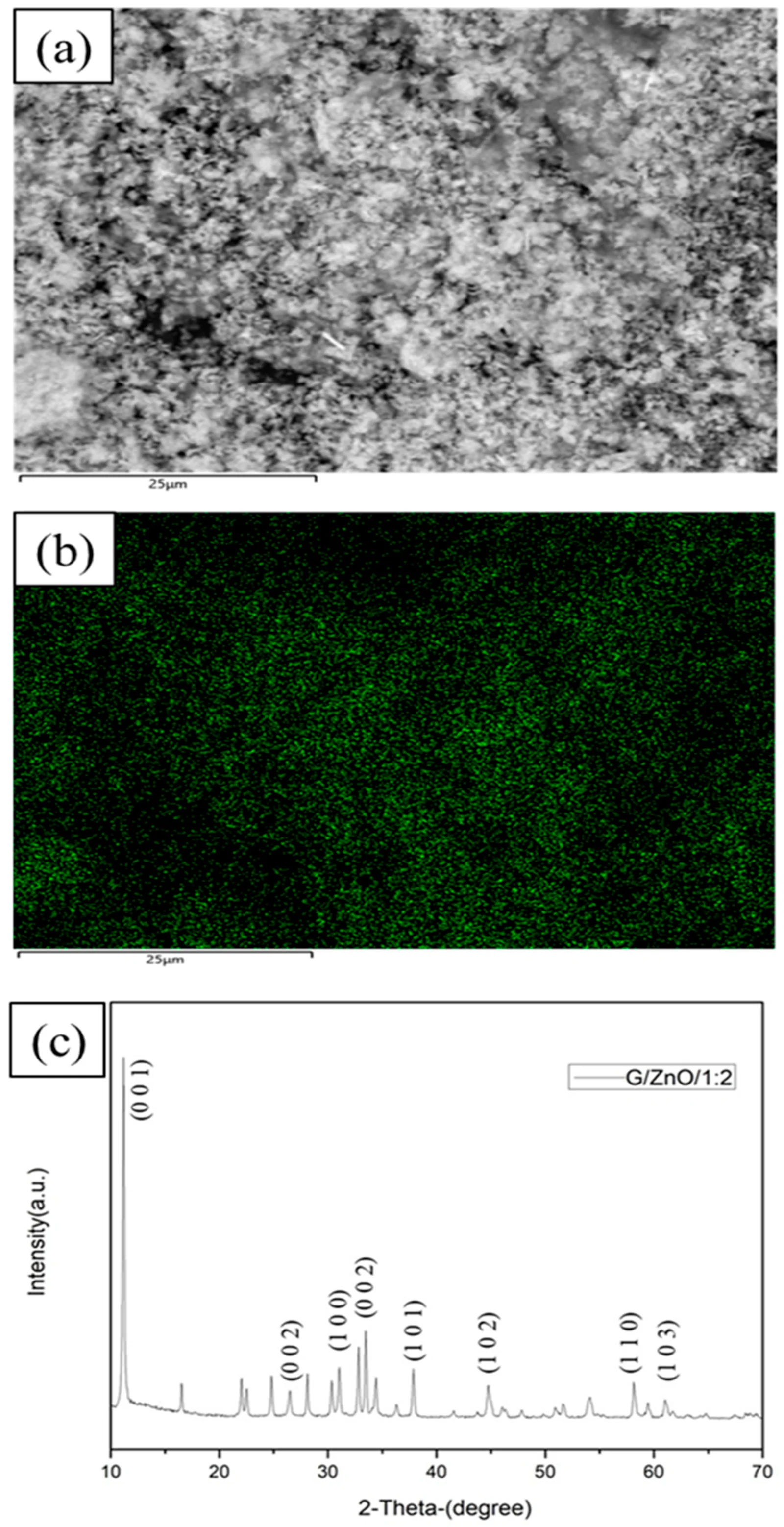
Characterization of the commercially supplied G/ZnO (1:2) hybrid filler used in this study: (**a**) morphological image, (**b**) Zn elemental mapping image, and (**c**) X-ray diffraction (XRD) pattern.

Figure 1 presents the morphological, elemental-mapping, and XRD characterization of the pristine commercial G/ZnO (1:2) hybrid filler. As shown in Figure 1a, the commercial G/ZnO hybrid filler exhibited an irregular and aggregated morphology rather than well-defined isolated particles. Zn elemental mapping (Figure 1b) showed that Zn-containing species were distributed throughout the examined region, while the XRD pattern (Figure 1c) further confirmed the presence of crystalline ZnO. However, these morphological and elemental-mapping results are insufficient to establish a specific nanoscale graphene–ZnO configuration, such as graphene-coated ZnO particles or a core–shell structure. Moreover, because of the pronounced aggregation of the commercial hybrid filler and the absence of DLS or TEM measurements, the primary particle-size distribution could not be quantitatively determined in the present study.

The G/ZnO material used in this study was commercially supplied under the designation of a graphene/ZnO hybrid filler with a nominal carbon-to-ZnO mass ratio of approximately 1:2. Additional morphological observation, Zn elemental mapping, and XRD analysis were performed to characterize the pristine hybrid filler. However, Raman spectroscopy and HRTEM were not performed; therefore, the number of graphene layers and defect density of the carbonaceous component could not be independently determined. Accordingly, the term “graphene” follows the supplier’s commercial product designation and should not be interpreted as independent experimental confirmation of strictly monolayer graphene.

### 3.2. Structural Characterization of PU Nanocomposites

It should be noted that polyurethane formulations containing graphene and ZnO individually at equivalent concentrations were not included in the present experimental design. Therefore, a synergistic effect between graphene and ZnO cannot be established from the present results. Accordingly, the observed changes in the structural and physicochemical properties are discussed as the overall effects associated with varying the loading of the commercially supplied G/ZnO hybrid filler within the BBD-containing PU matrix.

**Figure 2:**
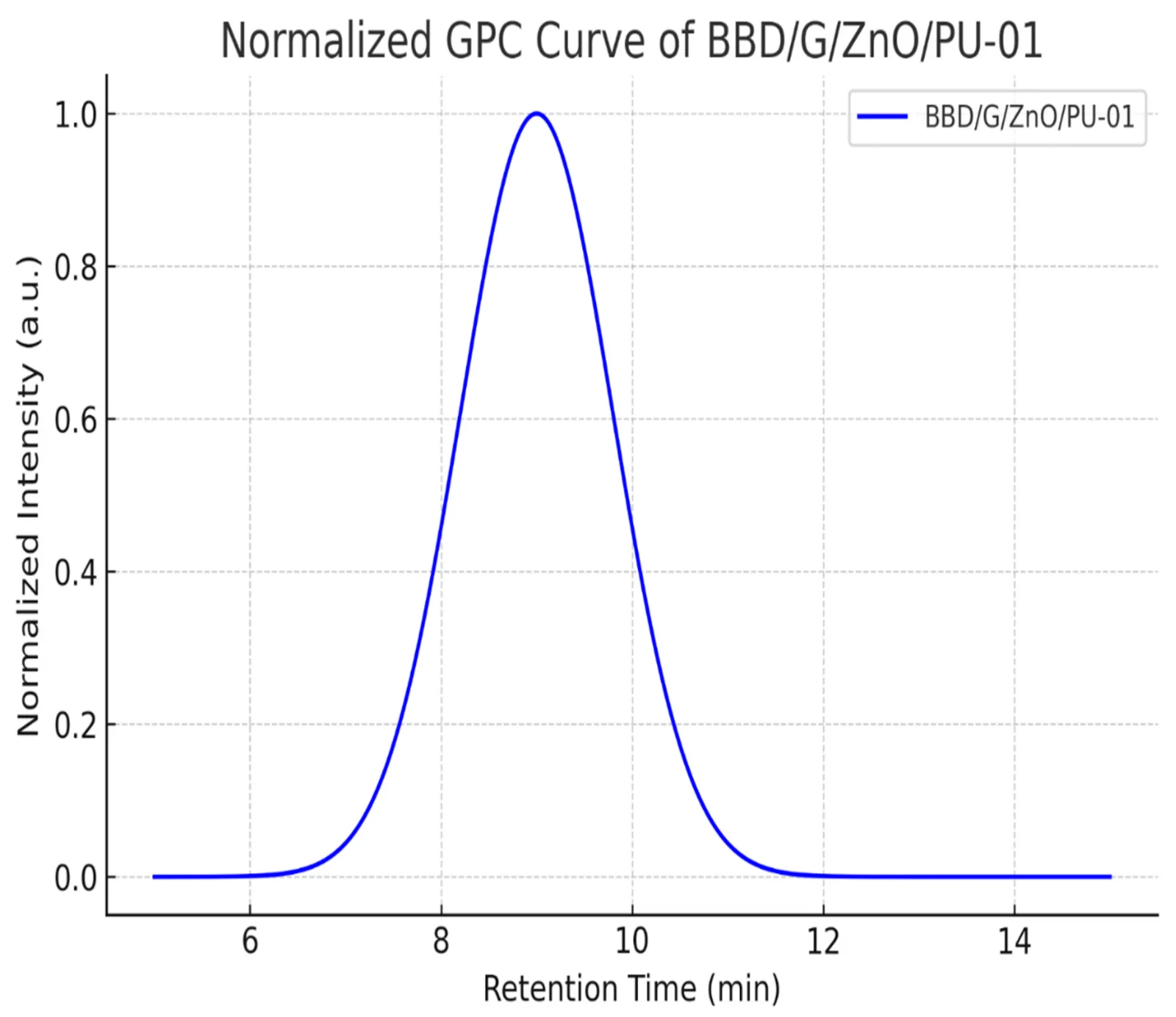
Gel permeation chromatography (GPC) chromatogram of the synthesized BBD/G/ZnO/PU-01 polyurethane.

The molecular-weight characteristics of the synthesized polyurethane were evaluated by gel permeation chromatography (GPC). Since the incorporation of G/ZnO nanohybrids may interfere with chromatographic analysis, the unfilled sample (BBD/G/ZnO/PU-01) was selected for measurement. The representative GPC chromatogram is shown in Figure 2, and the corresponding molecular-weight data are summarized in Table 3.

The synthesized polyurethane exhibited a number-average molecular weight (Mn) of (2.41 × 10^4^) g·mol^−1^ and a weight-average molecular weight (Mw) of (4.59 × 10^4^) g·mol^−1^, with a dispersity (PDI = Mw/Mn) of 1.90. The chromatogram displayed a single dominant peak without obvious low-molecular-weight fractions, indicating successful polymerization and the absence of significant amounts of residual oligomers. The obtained dispersity is consistent with the molecular-weight distribution commonly observed in conventional step-growth polyurethane polymerization and suggests that the polymerization proceeded efficiently to produce a stable polyurethane matrix [30,31].

These GPC results, together with the disappearance of the characteristic isocyanate absorption band in the FTIR spectra, further confirm the successful formation of the polyurethane network [30,31,32]. The resulting polyurethane matrix possessed an appropriate molecular weight for subsequent incorporation of G/ZnO nanohybrids and for evaluating their effects on the thermal, mechanical, surface, and antibacterial properties.

### 3.3. Fourier Transform Infrared Spectroscopy (FTIR) Analysis

**Figure 3:**
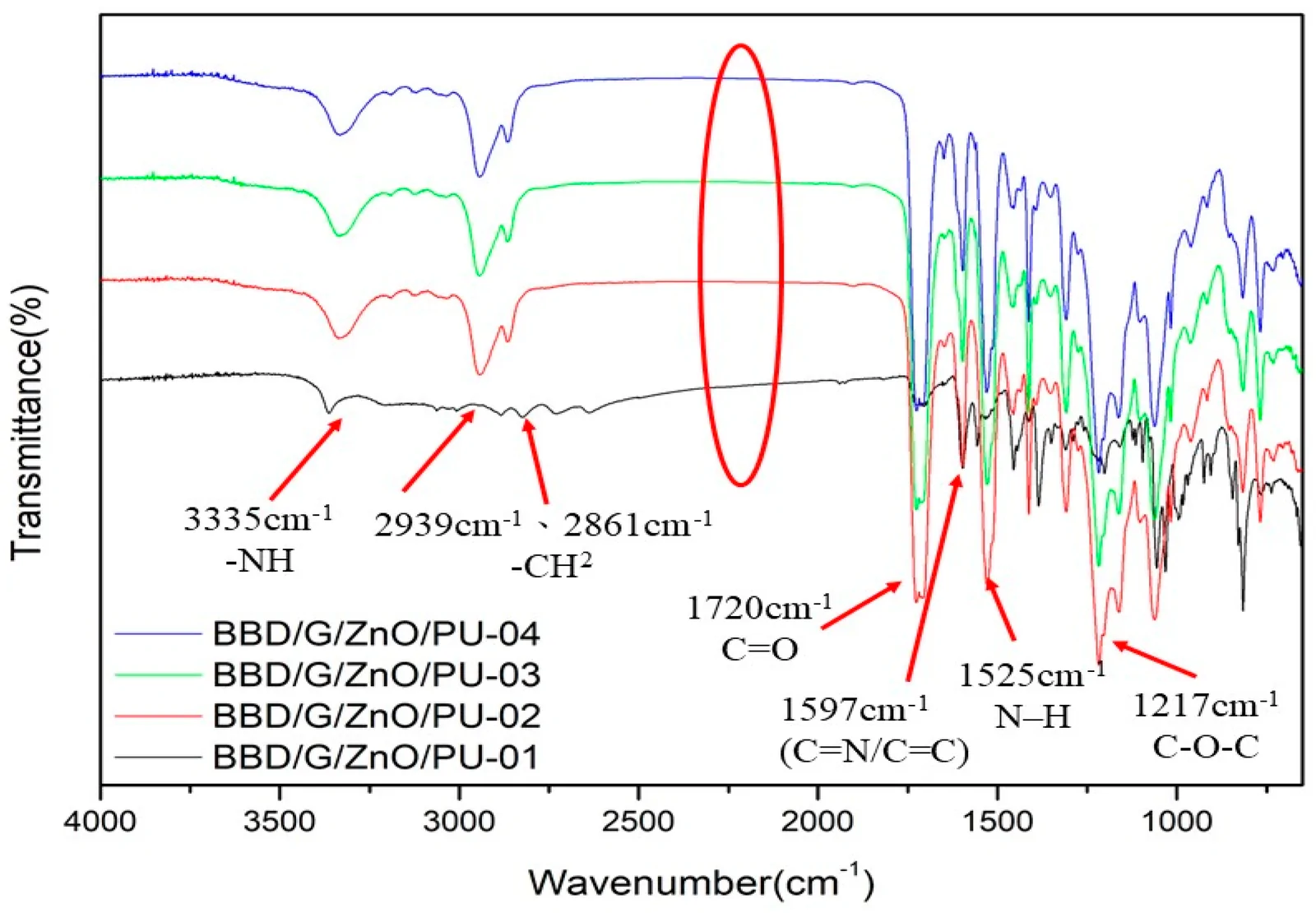
Fourier transform infrared (FTIR) spectra of BBD/G/ZnO/PUs. (The red circle highlights the region at approximately 2270 cm^−1^, where no detectable characteristic NCO absorption band was observed).

The chemical structures and possible intermolecular interactions of the BBD/G/ZnO/PU nanocomposites were investigated by Fourier transform infrared spectroscopy (FTIR), and the corresponding spectra are presented in Figure 3. All samples exhibited the characteristic absorption bands of segmented polyurethane, confirming the formation of the polyurethane structure after the two-step polymerization process. The broad absorption band observed at 3320–3400 cm^−1^ is assigned to the N–H stretching vibration of urethane groups (amide A), whereas the strong absorption band located near 1720 cm^−1^ corresponds to the C=O stretching vibration of urethane carbonyl groups. In addition, the absorption bands at approximately 1530–1540 cm^−1^ are attributed to the coupled N–H bending and C–N stretching vibrations of urethane linkages (amide II). Notably, the characteristic absorption band of the isocyanate group (–NCO) at approximately 2270 cm^−1^ was absent from the spectra, indicating the consumption of isocyanate groups during polyurethane formation.

These characteristic FTIR features are consistent with those reported for segmented polyurethane systems and polyurethane nanocomposites containing graphene- or ZnO-based fillers [4,7,9,15,21,31,32]. Hydrogen bonding involving urethane groups is known to influence intermolecular interactions and hard-segment organization in segmented polyurethane systems [4,9,31,32]. However, the present FTIR spectra were not quantitatively deconvoluted to distinguish free and hydrogen-bonded carbonyl or N–H species; therefore, changes in the extent of hydrogen bonding cannot be quantitatively established from the present results.

Following the incorporation of the G/ZnO hybrid filler, slight peak broadening and subtle intensity variations were observed in the N–H and C=O absorption regions, while no new absorption bands were detected. These spectral changes suggest variations in the local chemical environment of the polyurethane matrix following G/ZnO incorporation. Similar changes in the N–H and C=O regions have been reported for polyurethane systems containing graphene- and ZnO-based fillers and have been associated with changes in intermolecular and polymer–filler interfacial interactions [15,21]. However, the observed spectral variations alone are insufficient to identify a specific interaction mechanism.

The BBD chain extender introduces rigid bipyridyl units and nitrogen-containing sites into the polyurethane structure. Previous studies have reported intermolecular interactions in pyridyl-containing polyurethane systems, as well as coordination-related interactions involving pyridyl groups and metal species in related systems [31,33]. Nevertheless, the FTIR results obtained in the present study do not provide direct evidence for specific π–π interactions, Zn–pyridyl coordination, or other specific interactions between the individual components of the commercial G/ZnO hybrid filler and the polyurethane matrix. Therefore, the observed spectral variations are interpreted conservatively as changes in the local intermolecular and interfacial environment associated with G/ZnO incorporation rather than as evidence of a specific interaction mechanism.

### 3.4. Energy Dispersive Spectroscopy (EDS)

**Figure 4:**
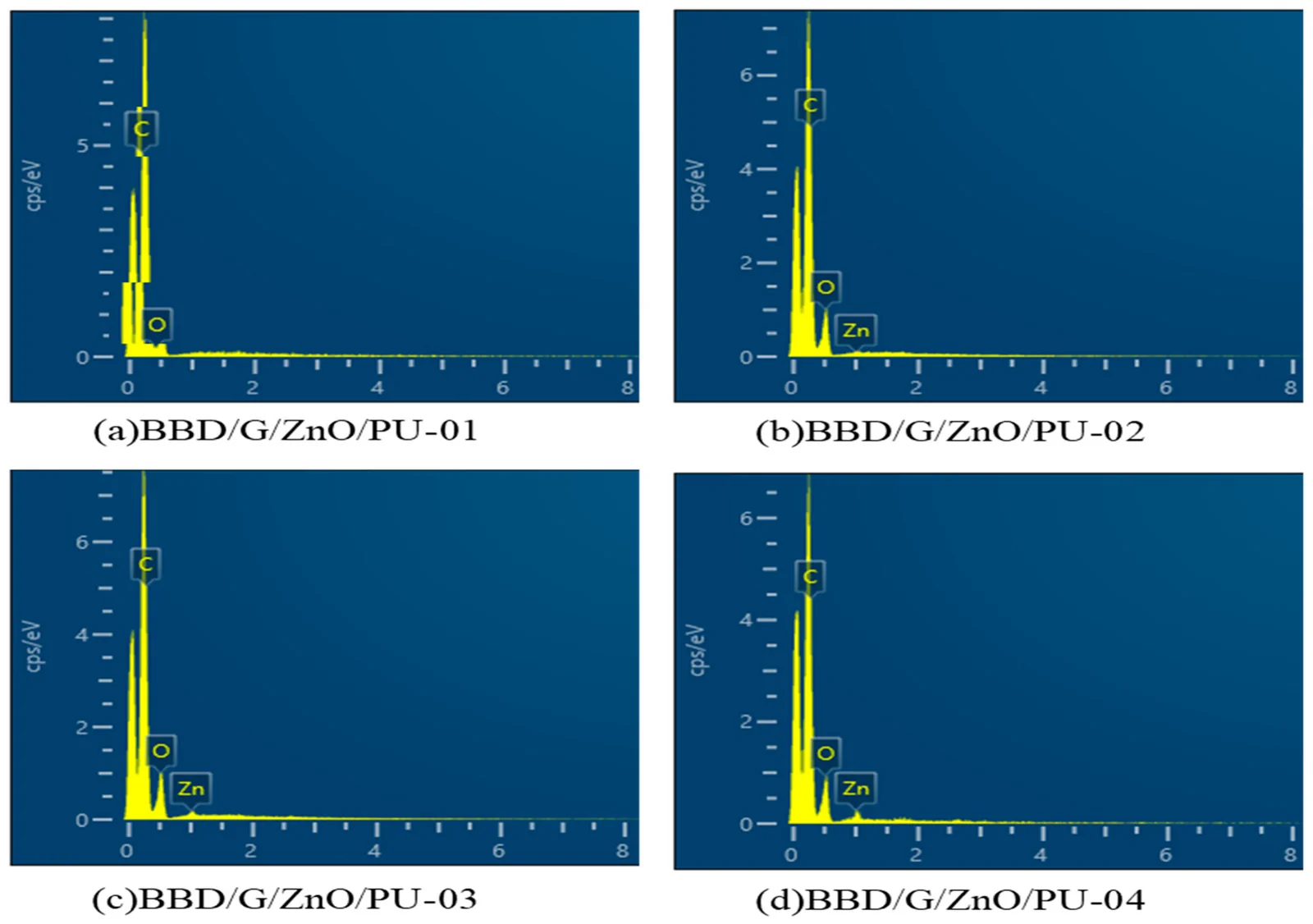
Energy-dispersive X-ray spectroscopy (EDS) analysis of BBD/G/ZnO/PUs.

**Figure 5:**
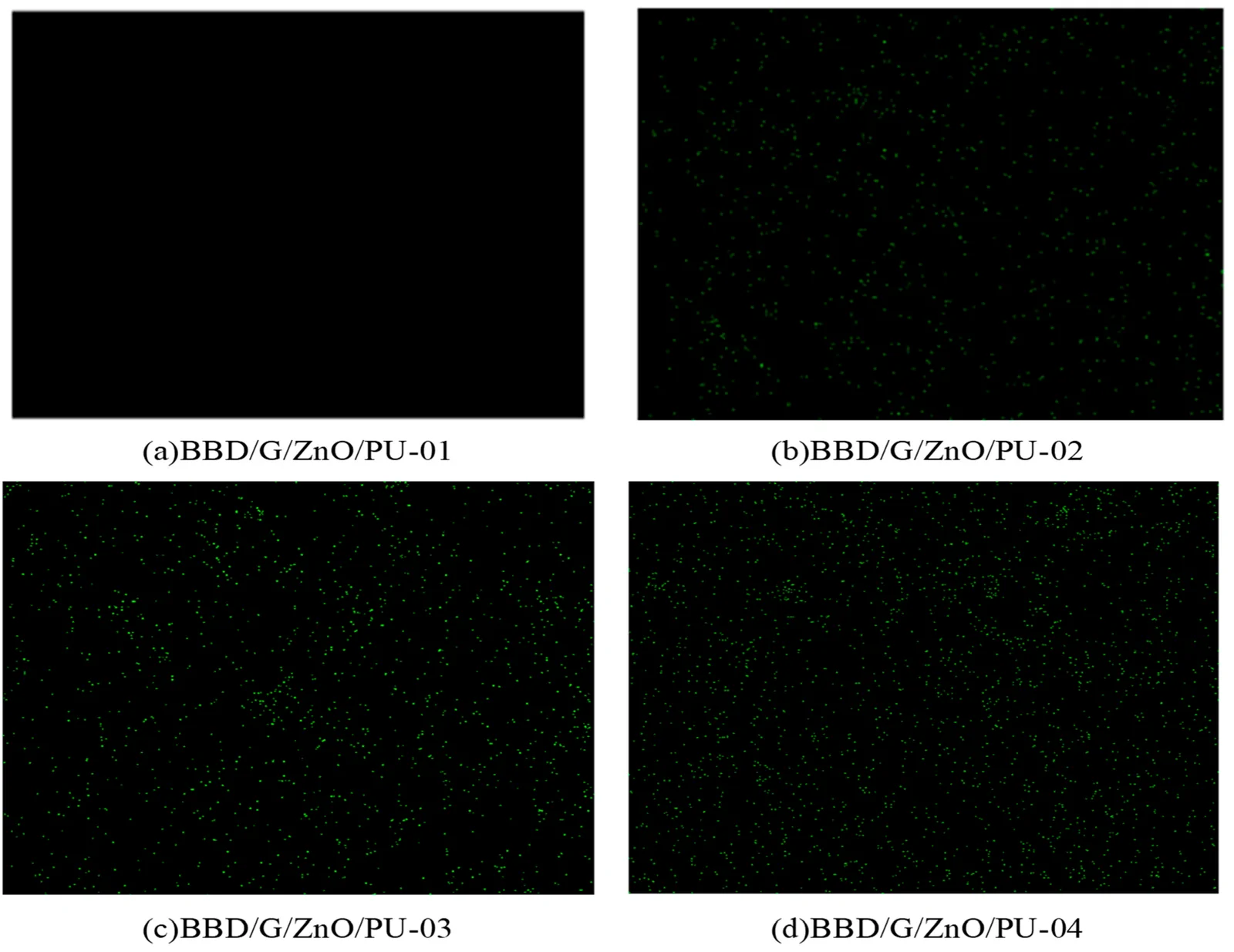
EDS elemental mapping of Zn in BBD/G/ZnO PUs.

EDS analysis was performed to verify the incorporation and spatial distribution of the Zn-containing component of the G/ZnO hybrid filler within the polyurethane matrix. The elemental compositions are summarized in Table 4, and the corresponding EDS spectra and elemental mapping images are presented in Figure 4 and Figure 5, respectively. No Zn signal was detected in the neat PU sample (PU-01), whereas the Zn content increased progressively with increasing G/ZnO loading from PU-02 to PU-04, confirming the incorporation of Zn-containing species into the polyurethane matrix [21,27,28,29]. As shown in Figure 5, Zn-containing regions were observed throughout the examined areas of the G/ZnO-containing samples. The intensity and spatial occurrence of the Zn signal generally increased with increasing G/ZnO loading, consistent with the corresponding EDS elemental compositions. These results indicate the spatial distribution of the Zn-containing component at the resolution of the SEM/EDS analysis. However, EDS mapping alone cannot determine the distribution of the carbonaceous/graphene component, the detailed graphene–ZnO nanoscale architecture, the primary particle-size distribution, or the presence or absence of nanoscale aggregation. Therefore, the EDS results are interpreted only as evidence for the presence and spatial distribution of Zn-containing regions within the examined areas and are not used to establish homogeneous nanoscale dispersion of the commercial G/ZnO hybrid filler.

### 3.5. X-Ray Diffraction (XRD)

**Figure 6:**
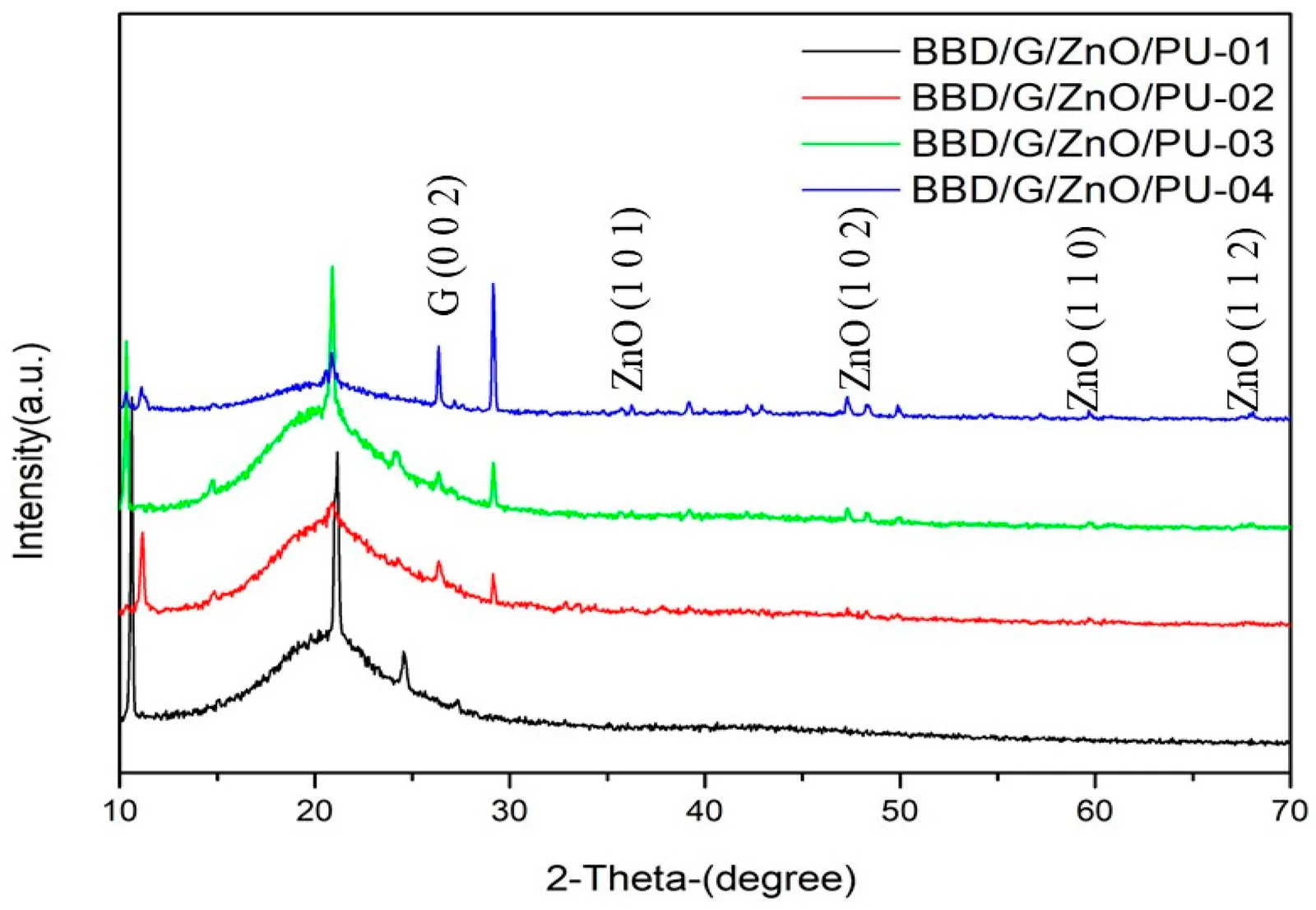
X-ray diffraction (XRD) patterns of BBD/G/ZnO/PUs.

The crystalline structures of the BBD/G/ZnO/PU nanocomposites were analyzed by X-ray diffraction (XRD), and the corresponding diffraction patterns are presented in Figure 6. The neat PU (PU-01) exhibited a broad diffraction halo at approximately 2θ = 19–25°, indicating the predominantly amorphous nature of the segmented polyurethane matrix, consistent with previous reports on polyurethane systems [4,31,32].

Following the incorporation of the G/ZnO hybrid filler, distinct diffraction peaks appeared at approximately 2θ = 36.2°, 47.5°, 56.6°, and 67.9°, corresponding to characteristic reflections of hexagonal wurtzite ZnO [21,27,28,29]. The intensities of these ZnO-related diffraction peaks generally increased with increasing G/ZnO loading, consistent with the increasing amount of the Zn-containing component in the nanocomposites. The characteristic ZnO diffraction peaks remained observable after composite preparation, indicating that the crystalline ZnO phase was retained within the polyurethane matrix [21,28,29].

Meanwhile, the broad amorphous halo of the polyurethane matrix remained evident after incorporation of the G/ZnO hybrid filler, indicating that the predominantly amorphous character of the polyurethane matrix was largely maintained. Thus, the XRD results primarily demonstrate the coexistence of a predominantly amorphous polyurethane matrix and crystalline ZnO-containing domains in the G/ZnO-containing samples. However, XRD alone cannot establish detailed changes in polyurethane microphase morphology or directly correlate these structural features with the thermal and mechanical properties of the nanocomposites. Therefore, the XRD results are interpreted primarily as evidence for the retention of the crystalline ZnO phase within the predominantly amorphous polyurethane matrix.

### 3.6. X-Ray Photoelectron Spectroscopy Analysis (XPS) Analysis

**Figure 7:**
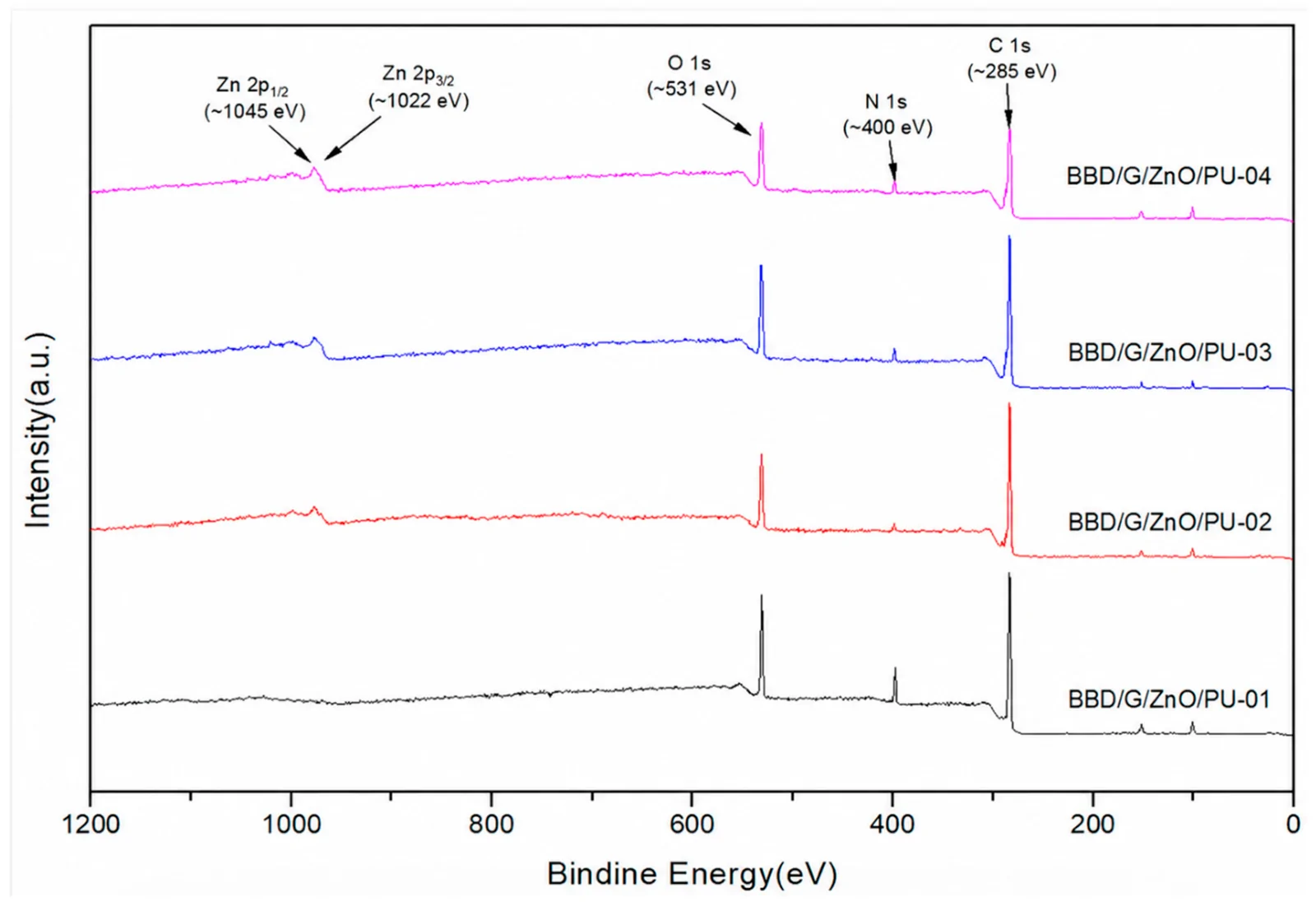
Surface elemental composition and XPS survey spectra of BBD/G/ZnO/PUs.

Figure 7 presents the XPS survey spectra of the BBD/G/ZnO/PU samples, and the corresponding surface elemental compositions are summarized in Table 5. The survey spectra show characteristic C 1s, O 1s, and N 1s signals for all samples, consistent with the elemental composition of the BBD-containing polyurethane matrix [31,44]. Similar C 1s, O 1s, and N 1s signals have previously been observed in XPS analyses of polyurethane systems [44]. In addition, Zn-related signals were detected in the G/ZnO-containing samples, whereas no Zn was detected in the control sample, BBD/G/ZnO/PU-01. These results provide surface-sensitive evidence for the incorporation of Zn-containing species into the BBD-containing polyurethane system [7,21].

As summarized in Table 5, BBD/G/ZnO/PU-01 exhibited surface atomic concentrations of 72.42 at.% C, 8.93 at.% O, and 18.65 at.% N, with no detectable Zn. Following the incorporation of the G/ZnO hybrid filler, the Zn content increased to 0.23, 0.29, and 0.82 at.% for BBD/G/ZnO/PU-02, BBD/G/ZnO/PU-03, and BBD/G/ZnO/PU-04, respectively. The overall increase in surface Zn concentration with increasing G/ZnO loading is consistent with the progressively higher amount of Zn-containing filler incorporated into the polyurethane formulations. The C, O, and N atomic concentrations also varied among the samples, reflecting changes in the surface composition of the multicomponent nanocomposite system. Because XPS is a surface-sensitive technique, these atomic percentages are interpreted as surface compositions rather than bulk elemental concentrations [44].

The XPS results provide complementary evidence that the incorporation of the G/ZnO hybrid filler alters the surface elemental composition and local chemical environment of the BBD-containing polyurethane matrix. Interfacial interactions between inorganic nanofillers and polyurethane can influence the phase organization and segmental mobility of polyurethane chains [7,21], while pyridyl-containing structures can also affect intermolecular interactions within polyurethane systems [31]. These considerations are consistent with the observed variations in glass transition temperature. However, the present XPS results do not provide sufficient evidence to assign these changes to a specific Zn–pyridyl coordination mechanism. Although coordination interactions have been reported in polyurethane systems containing coordination-capable functional groups [33], assignment of a specific coordination mechanism requires appropriate chemical-state evidence and controls. Accordingly, the present XPS results are interpreted conservatively as evidence of changes in surface elemental composition and local chemical environment associated with increasing G/ZnO loading, rather than as direct proof of a specific Zn–pyridyl coordination interaction.

### 3.7. Thermogravimetric Analysis (TGA)

**Figure 8:**
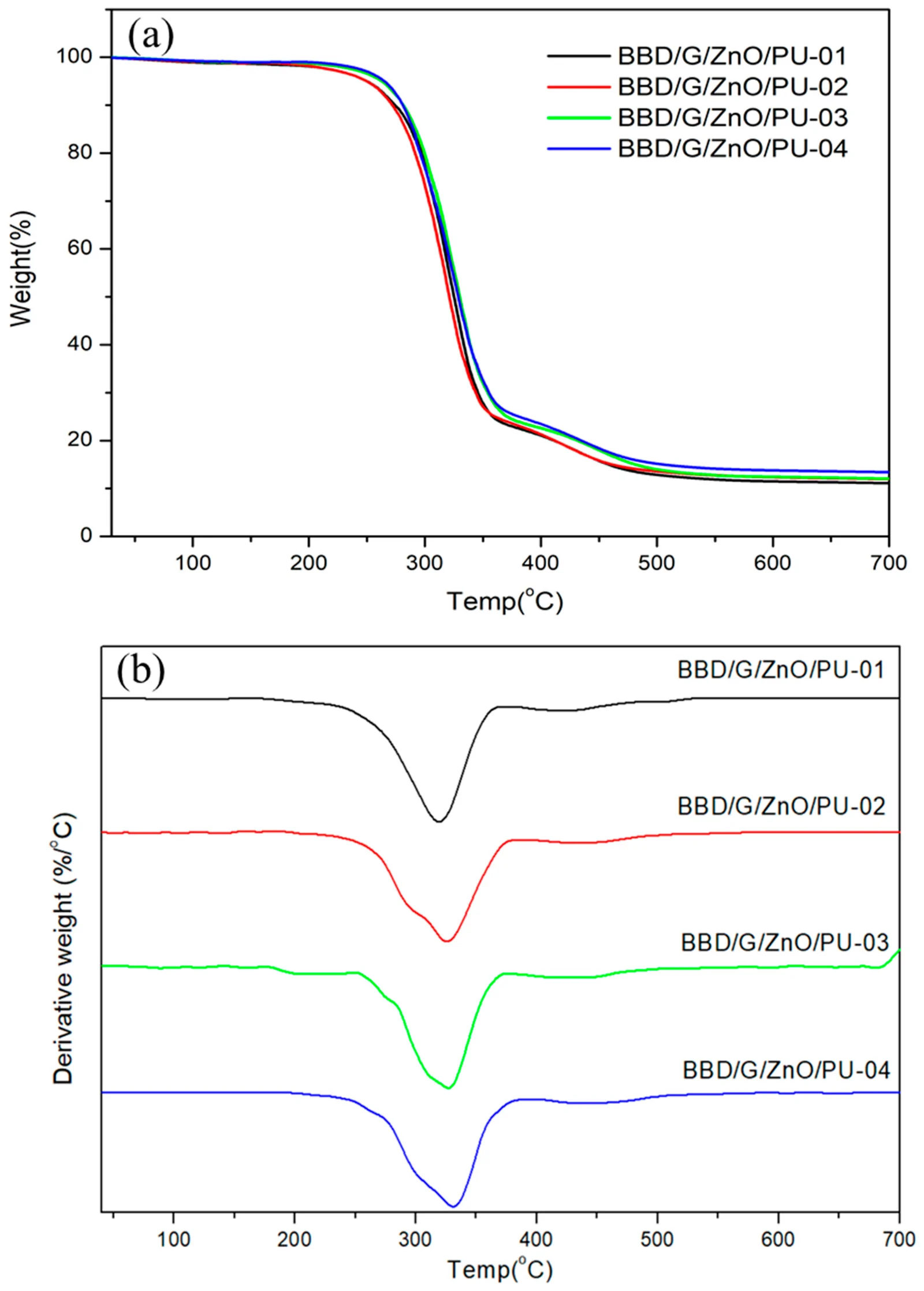
Thermal degradation behavior of BBD/G/ZnO/PUs: (**a**) TGA curves and (**b**) DTG curves.

The thermal degradation behavior of the BBD/G/ZnO/PU nanocomposites was evaluated by thermogravimetric analysis (TGA) under a continuous nitrogen atmosphere. The TGA and corresponding derivative thermogravimetric (DTG) curves are presented in Figure 8a,b, respectively, and the thermal degradation parameters are summarized in Table 6. The onset degradation temperature (*T*onset) gradually increased from 260.8 °C for BBD/G/ZnO/PU-01 to 264.8 °C for BBD/G/ZnO/PU-04 with increasing G/ZnO content. The DTG curves further showed that the temperature corresponding to the maximum degradation rate (*T*max) increased from 318.8 °C for BBD/G/ZnO/PU-01 to 331.3 °C for BBD/G/ZnO/PU-04. In addition, the residual mass at 700 °C increased from 11.0% to 13.3%. These results indicate modest, composition-dependent changes in the thermal degradation behavior of the BBD-containing PU system following the incorporation of G/ZnO.

The gradual increases in *T*onset and *T*max may be associated with the presence of the G/ZnO hybrid filler within the polyurethane matrix. Carbonaceous components such as graphene-based materials can provide a physical barrier that may hinder heat and mass transport and retard the diffusion of volatile degradation products, while thermally stable inorganic ZnO can contribute to the residual inorganic fraction during thermal decomposition [15,18,19,21]. However, the increase in *T*onset from 260.8 to 264.8 °C corresponds to only 4.0 °C and is therefore relatively modest from a practical standpoint. Similarly, the increase in residual mass at 700 °C from 11.0% to 13.3% is limited and may partly reflect the increasing amount of thermally stable inorganic/carbonaceous components introduced with increasing G/ZnO content rather than an intrinsic improvement in the thermal stability of the polyurethane matrix. Similar changes in thermal degradation behavior have been reported for polyurethane systems containing graphene- and ZnO-based fillers [7,15,18,19,21].

Interfacial interactions between the polyurethane matrix and the incorporated filler may also influence polymer-chain mobility and thermal degradation behavior [15,18,19,21,32]. In addition, the pyridyl-containing BBD structure may contribute to intermolecular interactions within the polyurethane matrix [31], while coordination-related interactions involving pyridyl groups and metal species have been reported in related systems [33]. However, the present thermal analysis does not provide direct evidence for such specific coordination interactions in the BBD/G/ZnO/PU system. Moreover, because separate PU/graphene and PU/ZnO control samples were not included, the individual contributions of graphene and ZnO, as well as any synergistic effect between these components, cannot be distinguished. Therefore, the observed changes in *T*onset, *T*max, and residual mass are interpreted conservatively as trends in thermal degradation behavior associated with increasing G/ZnO content rather than as evidence of a specific interfacial mechanism or a substantial or synergistic enhancement in thermal stability. TGA measurements were performed once for each composition (*n* = 1). Therefore, statistically derived experimental uncertainties, such as standard deviations or error bars, cannot be reported. Accordingly, the thermal degradation parameters presented in Table 6 should be regarded as values obtained from single measurements, and the observed trends should be interpreted within this experimental limitation.

### 3.8. Differential Scanning Calorimeter (DSC)

**Figure 9:**
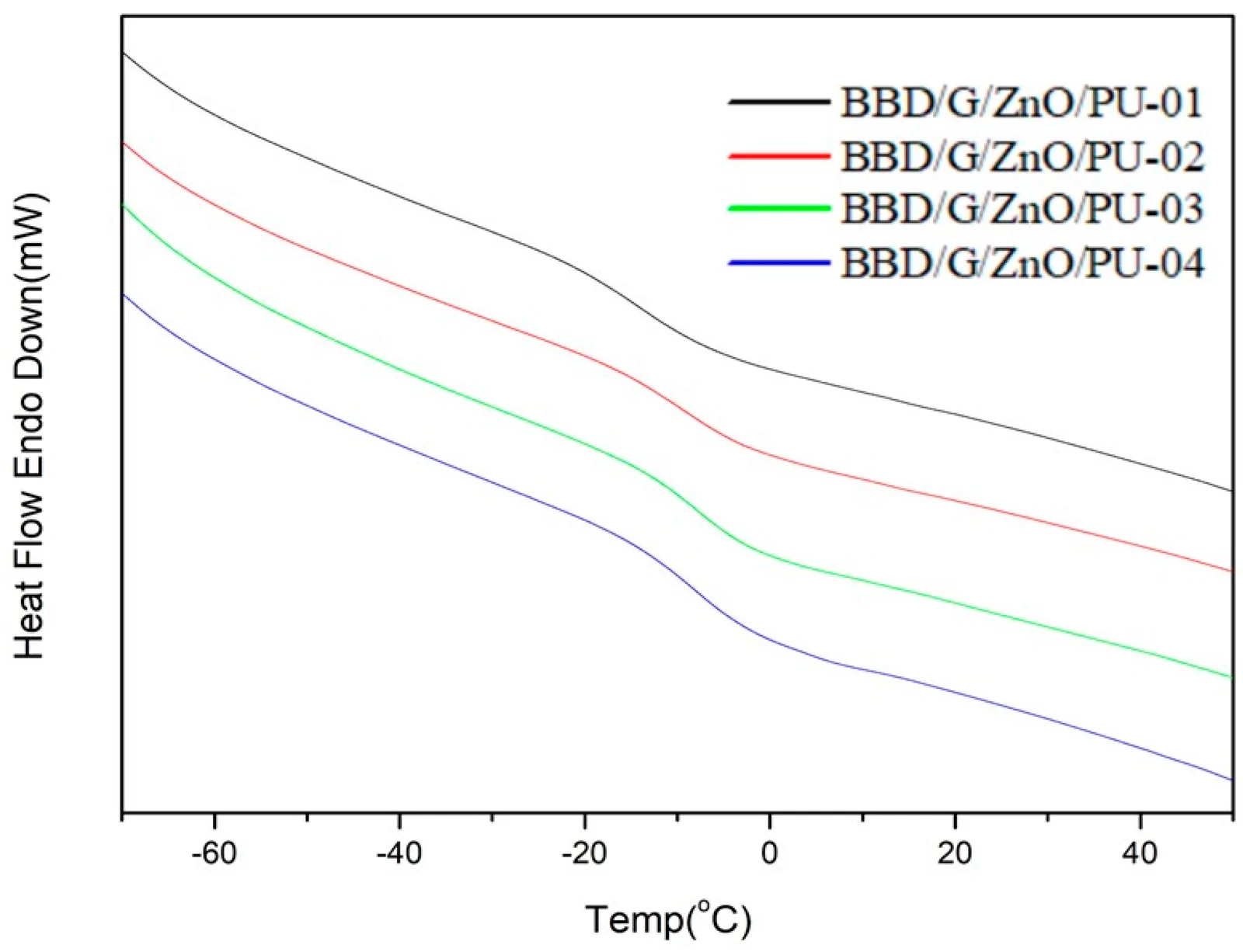
Differential scanning calorimetry (DSC) thermograms of BBD/G/ZnO/PUs.

The thermal transition behavior and segmental dynamics of the BBD/G/ZnO/PU nanocomposites were investigated by differential scanning calorimetry (DSC). The corresponding thermograms are presented in Figure 9, and the glass transition temperatures (Tg) of the soft segments are summarized in Table 7. The Tg values gradually increased from −13.91 °C for neat PU (PU-01) to −7.97 °C for PU-04 with increasing G/ZnO loading, indicating changes in the segmental mobility of the polyurethane chains. Similar increases in Tg have been reported for graphene- and ZnO-containing polyurethane nanocomposites and have been associated with filler-induced changes in polymer-chain mobility and polymer–filler interactions [4,15,18,30,32].

The observed Tg shift may be associated with changes in the local interfacial environment following incorporation of the G/ZnO hybrid filler. Graphene- and ZnO-containing polyurethane systems have previously exhibited changes in segmental relaxation behavior after filler incorporation [15,18,21,32], while the pyridyl-containing BBD structure may also influence intermolecular interactions within the polyurethane matrix [31]. However, because the BBD content was maintained constant and separate graphene- and ZnO-containing control samples were not included, the individual contributions of BBD, graphene, and ZnO to the observed Tg changes cannot be distinguished from the present results.

The XPS results provide complementary evidence of changes in the surface elemental composition and local chemical environment of the BBD/G/ZnO/PU system. These observations are consistent with matrix–filler interfacial interactions that may influence segmental mobility. However, the present results do not provide sufficient evidence to assign the Tg changes to a specific coordination mechanism. Therefore, the observed Tg variations are interpreted conservatively as reflecting changes in the local interfacial environment and polymer-chain mobility associated with increasing G/ZnO loading.

### 3.9. Dynamic Mechanical Analysis (DMA)

**Figure 10:**
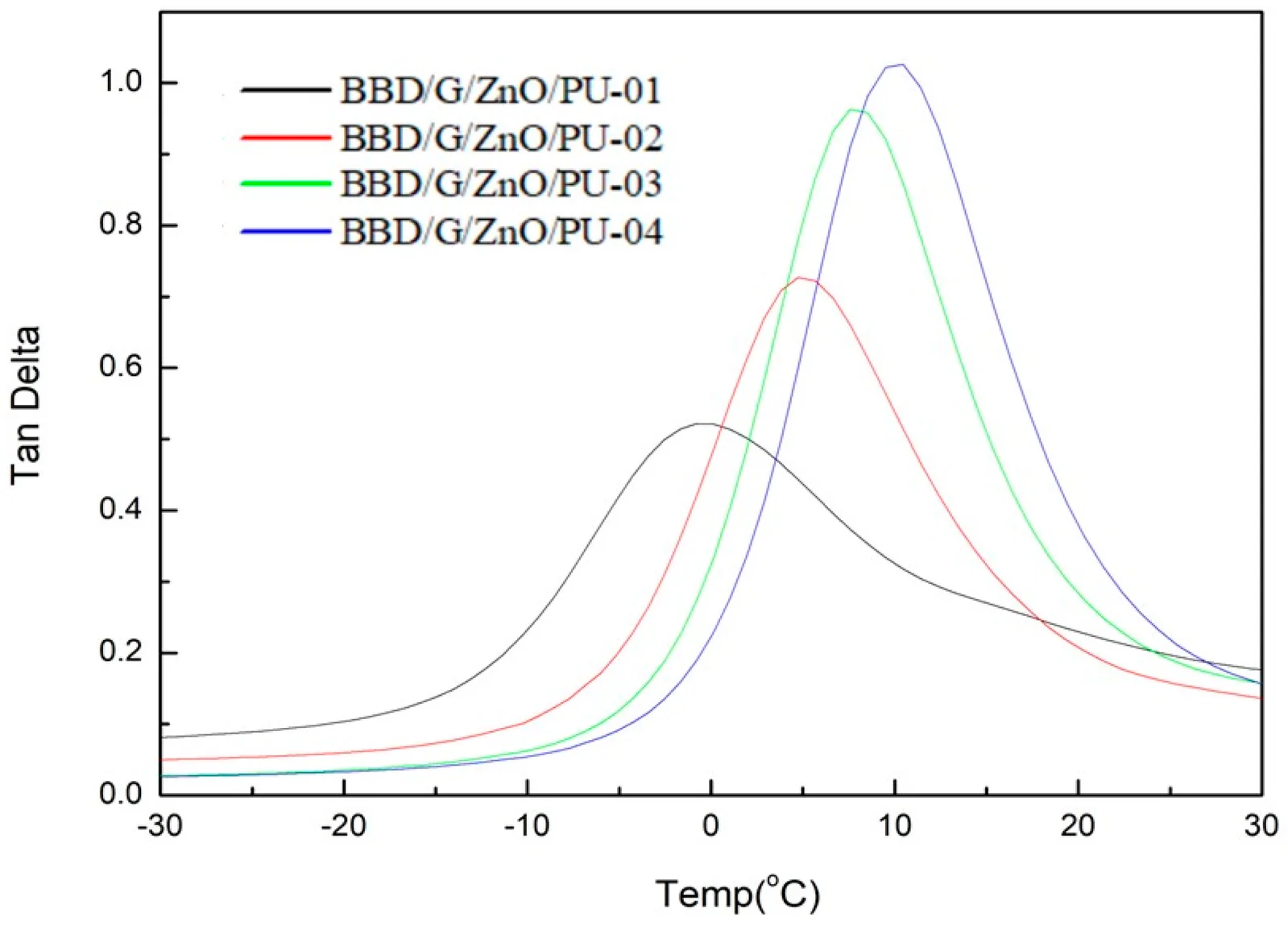
Dynamic mechanical analysis (DMA) tan δ curves of BBD/G/ZnO/PUs.

**Figure 11:**
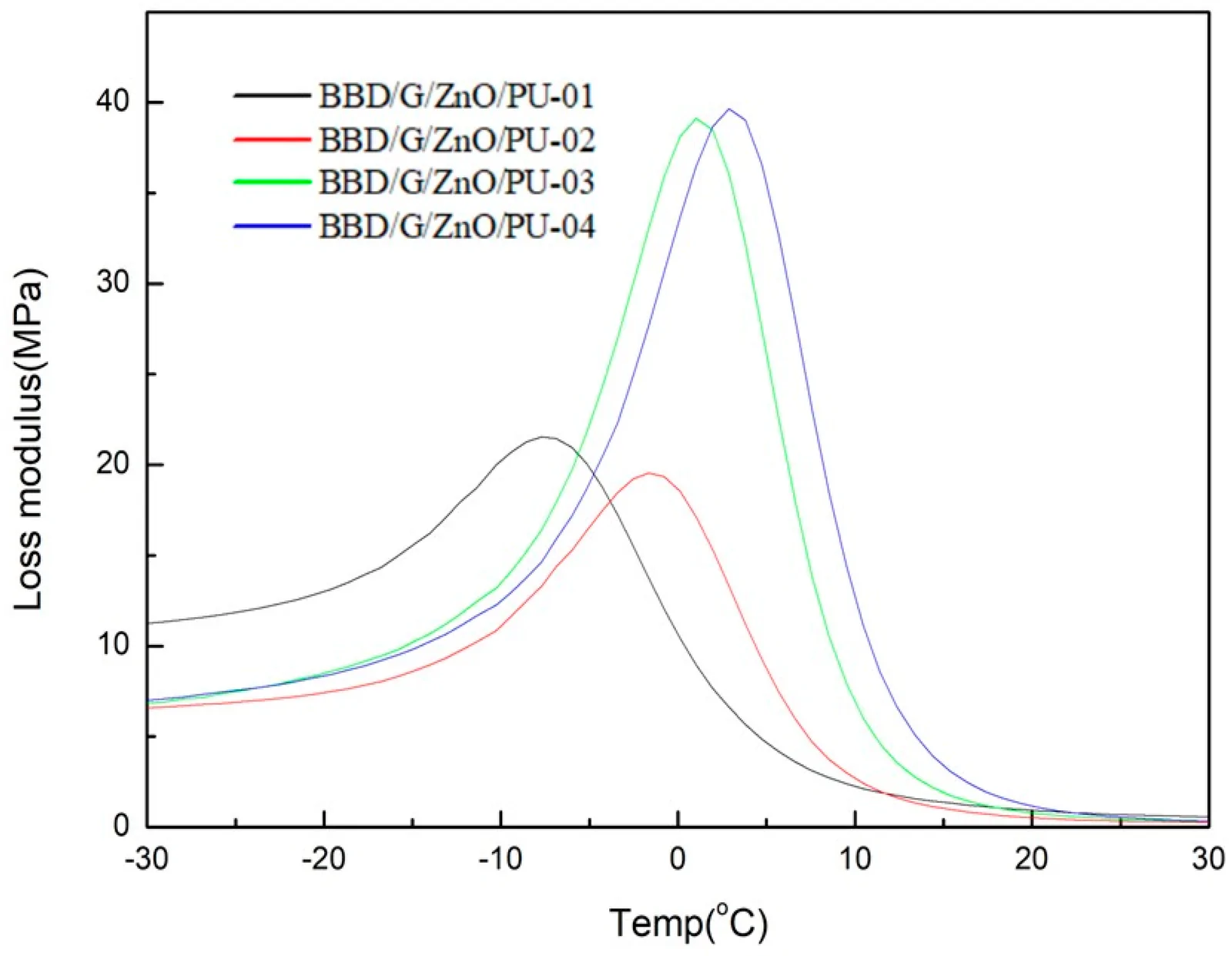
Dynamic mechanical analysis (DMA) loss modulus (E″) curves of BBD/G/ZnO/PUs.

**Figure 12:**
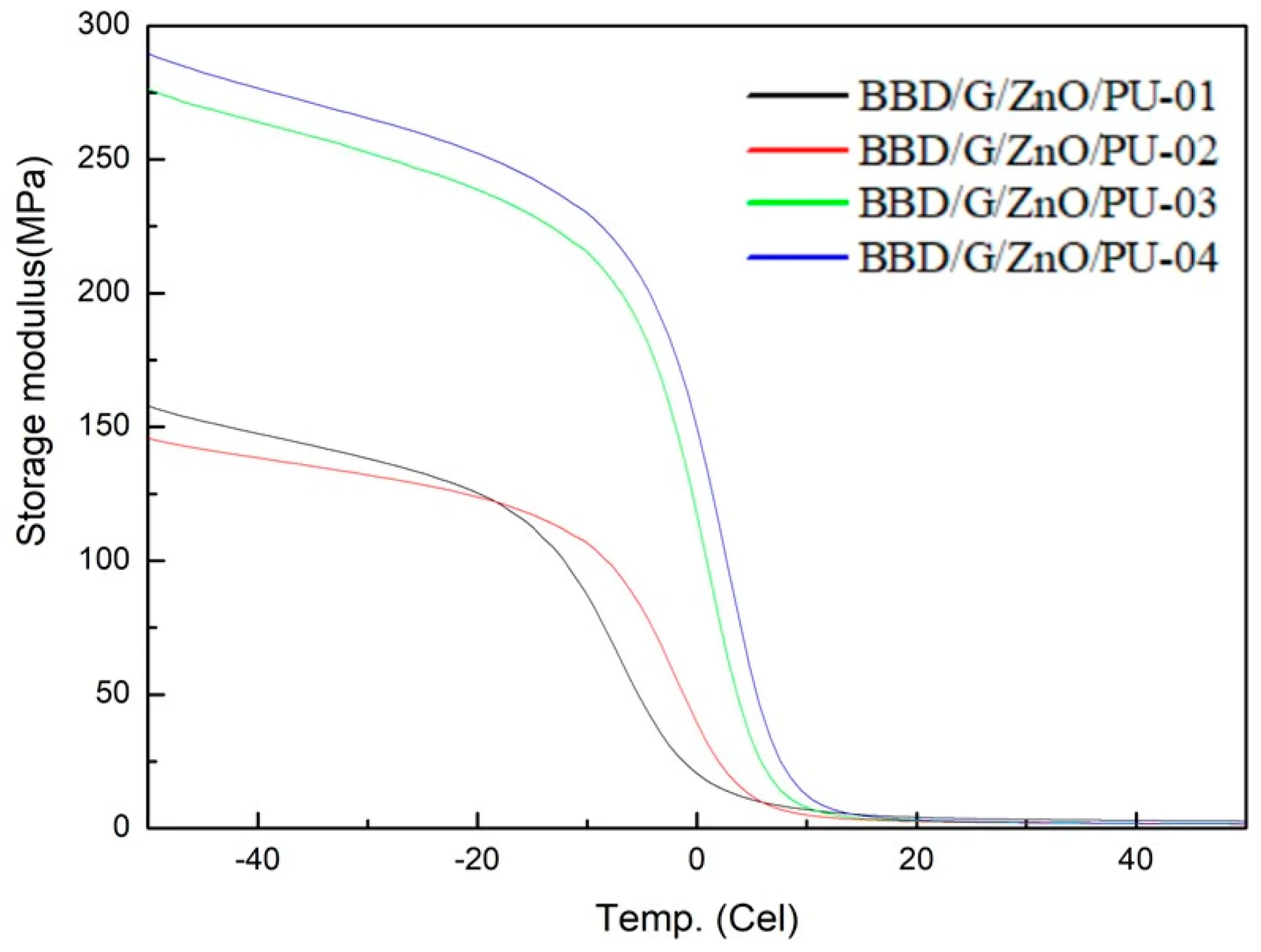
Dynamic mechanical analysis (DMA) storage modulus (E′) curves of BBD/G/ZnO/PUs.

The viscoelastic properties and relaxation behavior of the BBD/G/ZnO/PU nanocomposites were investigated by dynamic mechanical analysis (DMA). The corresponding thermomechanical profiles are presented in Figure 10, Figure 11 and Figure 12, and the extracted parameters are summarized in Table 8. Both the loss modulus (E″) peak temperature and the damping factor (tan δ) peak temperature shifted progressively toward higher temperatures with increasing G/ZnO loading, indicating composition-dependent changes in the segmental mobility and relaxation behavior of the polyurethane chains. In addition, the storage modulus (E′) increased with increasing G/ZnO loading over the investigated temperature range, indicating an increase in the stiffness of the polyurethane nanocomposites. Similar increases in storage modulus and shifts in glass-transition-related relaxation temperatures have been reported for graphene- and ZnO-containing polyurethane nanocomposites and have been associated with filler-induced changes in polymer-chain mobility and polymer–filler interactions [4,15,18,21,30,32].

The observed changes in the DMA response may be associated with the incorporation of the G/ZnO hybrid filler and the resulting changes in the local polymer–filler interfacial environment. Graphene- and ZnO-containing polyurethane nanocomposites have previously exhibited reinforcing and chain-confinement effects that can influence storage modulus and relaxation behavior [15,18,21]. The pyridyl-containing BBD structure may also influence intermolecular interactions within the polyurethane matrix [31]. However, because the BBD content was maintained constant and separate graphene- and ZnO-containing control samples were not included, the individual contributions of BBD, graphene, and ZnO, as well as any synergistic effect between graphene and ZnO, cannot be distinguished from the present DMA results. Therefore, the observed changes are conservatively interpreted as the overall effects associated with increasing the loading of the commercially supplied G/ZnO hybrid filler within the BBD-containing polyurethane matrix.

The maximum damping factor (tan δmax) increased with increasing G/ZnO loading, while the tan δ peak shifted toward higher temperatures. These changes indicate composition-dependent variations in the relaxation and energy-dissipation behavior of the polyurethane nanocomposites. The DMA results are generally consistent with the DSC results, which also showed a shift in glass-transition behavior with increasing G/ZnO loading. Taken together, the DSC and DMA results indicate that increasing the G/ZnO hybrid-filler loading alters the segmental mobility, relaxation behavior, and thermomechanical response of the BBD-containing polyurethane system. However, the present results do not provide sufficient evidence to assign these changes to a specific molecular interaction or interfacial mechanism.

### 3.10. Stress–Strain Testing

**Figure 13:**
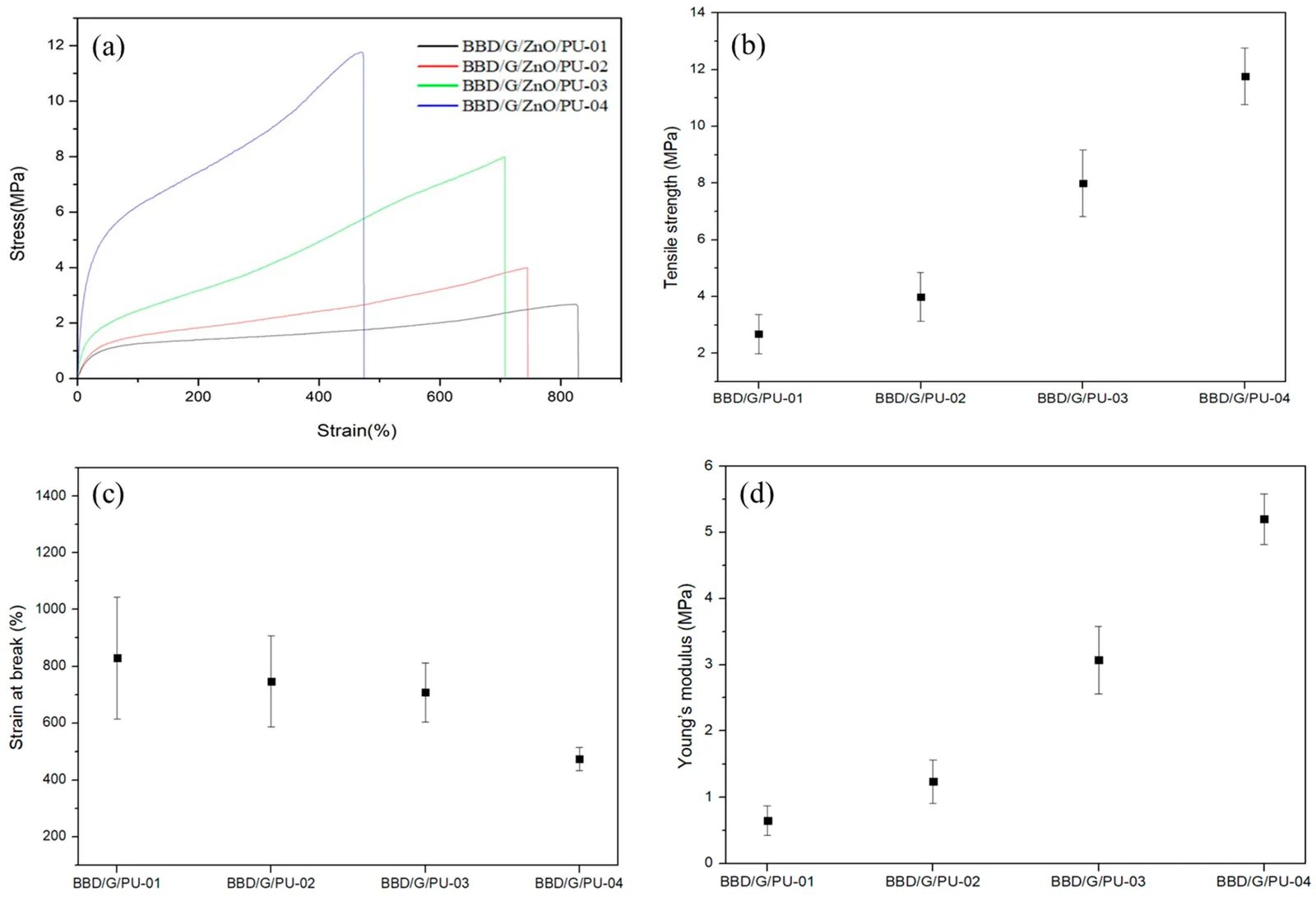
Mechanical properties of BBD/G/ZnO/PUs: (**a**) representative stress–strain curves, (**b**) tensile strength, (**c**) elongation at break, and (**d**) Young’s modulus. Data in (**b**–**d**) are presented as mean ± SD (*n* = 3).

The tensile properties of the BBD/G/ZnO/PU nanocomposites were evaluated at ambient temperature in accordance with the ASTM D882 standard. Figure 13 presents the representative stress–strain curves and summarizes the tensile strength, elongation at break, and Young’s modulus of the BBD/G/ZnO/PU samples. The quantitative mechanical properties are presented as the mean ± standard deviation (SD) based on three replicate specimens (*n* = 3), and the corresponding values are summarized in Table 9. The tensile strength increased from 2.68 MPa for BBD/G/ZnO/PU-01 to 11.76 MPa for BBD/G/ZnO/PU-04 with increasing G/ZnO content. The Young’s modulus also increased from 0.65 to 5.20 MPa over the same composition range. These results indicate an increase in the strength and stiffness of the polyurethane matrix with increasing G/ZnO loading. Similar trends in mechanical reinforcement have been reported for polyurethane nanocomposites containing graphene- and ZnO-based fillers, where polymer–filler interactions and the reinforcing characteristics of the incorporated fillers can influence stress transfer and mechanical behavior [4,15,18,21,45].

The observed increases in tensile strength and Young’s modulus may be associated with the reinforcing contribution of the G/ZnO hybrid filler and its interactions with the polyurethane matrix. Graphene-based components, owing to their high aspect ratio and large specific surface area, can contribute to load transfer within polymer composites, while ZnO particles may interact with polar groups in polyurethane and thereby influence the mechanical response of the composite [15,16,18,21,22,23]. In addition, the pyridyl-containing BBD structure may contribute to intermolecular interactions within the polyurethane matrix [31], and coordination-related interactions involving pyridyl groups and metal species have been reported in related systems [33]. However, the present mechanical data do not directly establish such specific coordination interactions in the BBD/G/ZnO/PU system. Furthermore, because separate PU/graphene and PU/ZnO control samples were not included, the individual contributions of graphene and ZnO and any synergistic effect between these components cannot be quantitatively distinguished. Therefore, the observed mechanical changes are conservatively attributed to the incorporation of the commercial G/ZnO hybrid filler into the BBD-containing polyurethane matrix rather than to a specifically demonstrated synergistic mechanism.

Conversely, the elongation at break decreased from 829% for BBD/G/ZnO/PU-01 to 474% for BBD/G/ZnO/PU-04 as the G/ZnO content increased. This trend is consistent with the behavior commonly observed in rigid-filler-containing polyurethane systems, in which the incorporation of reinforcing domains can restrict polymer-chain mobility and reduce deformability during tensile loading [15,18,32]. Thus, increasing G/ZnO content was accompanied by higher tensile strength and Young’s modulus but lower elongation at break, indicating a shift toward a stronger and stiffer, but less extensible, mechanical response. Because no inferential statistical significance testing was performed in the present study, these differences are discussed as experimentally observed trends rather than statistically significant differences. Overall, the mechanical results demonstrate composition-dependent changes in the tensile behavior of the BBD/G/ZnO/PU system [15,31,32,33,44].

### 3.11. Water Contact Angle (WCA)

**Figure 14:**
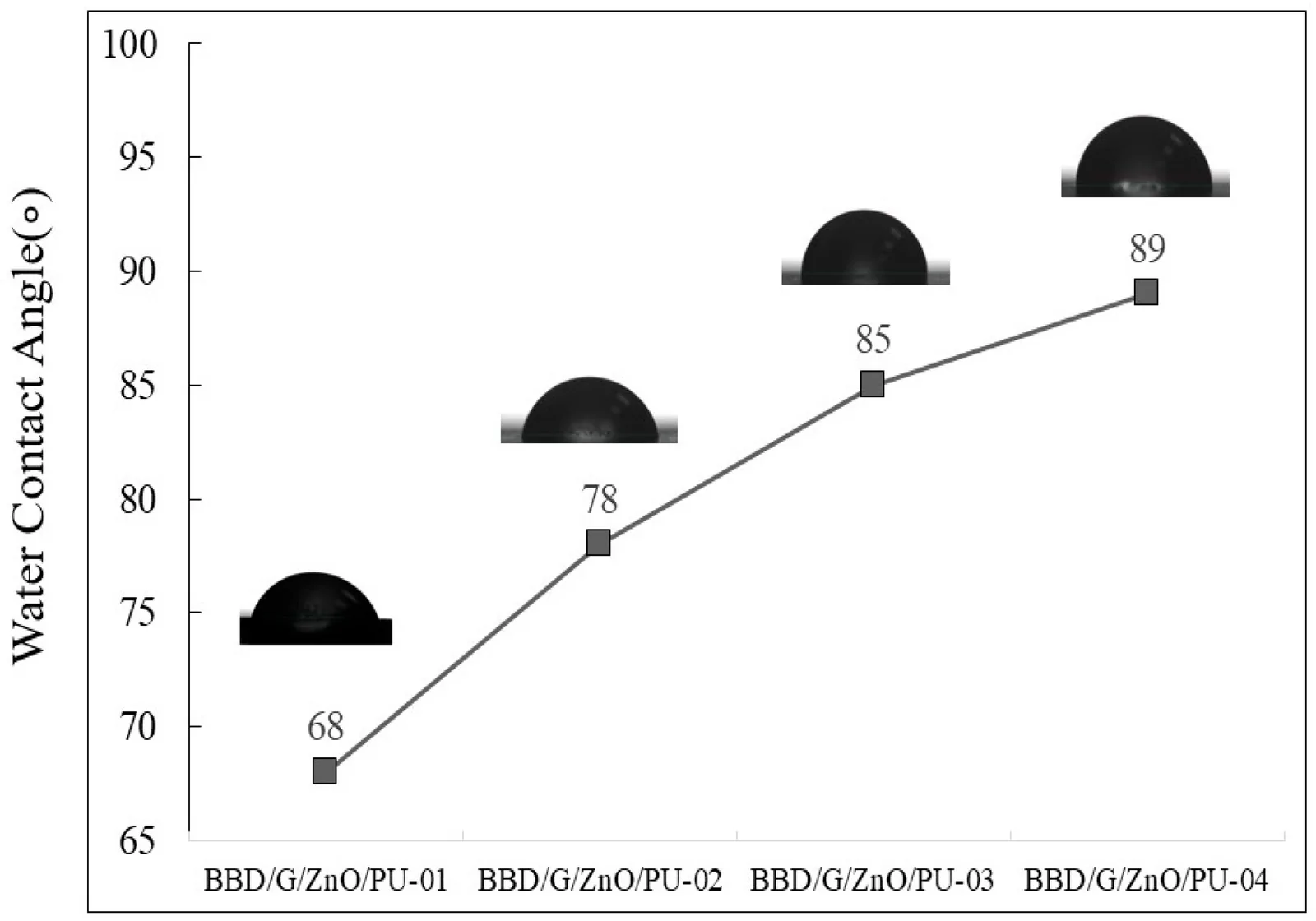
Water contact angle (WCA) of BBD/G/ZnO/PUs. (The images above the contact angle values show representative water droplet profiles for the corresponding samples).

The surface wettability of the BBD/G/ZnO/PU nanocomposites was evaluated by static water contact angle (WCA) measurements, and the corresponding results are presented in Figure 14. The water contact angle increased progressively from approximately 68° for the neat PU (PU-01) to approximately 89° for the composite containing 2.0 wt.% G/ZnO (PU-04), indicating a gradual decrease in apparent surface wettability with increasing G/ZnO hybrid-filler loading. Similar changes in water contact angle have been reported for graphene- and ZnO-modified polyurethane systems and have been associated with changes in surface chemical composition and/or surface morphology following filler incorporation [15,21,27,37].

In the present study, the increase in water contact angle may be associated with changes in the surface chemical environment and/or surface morphology following incorporation of the G/ZnO hybrid filler. The XPS results provide complementary evidence of changes in the surface elemental composition of the BBD/G/ZnO/PU nanocomposites after G/ZnO incorporation. However, because quantitative surface-roughness and surface-energy measurements were not performed, the relative contributions of surface chemical composition and morphology to the observed wettability changes cannot be independently determined from the present results. Therefore, the increase in water contact angle is interpreted conservatively as an overall change in the apparent surface wettability associated with increasing G/ZnO hybrid-filler loading rather than as evidence of a specific wetting mechanism.

### 3.12. Antibacterial Activity

**Figure 15:**
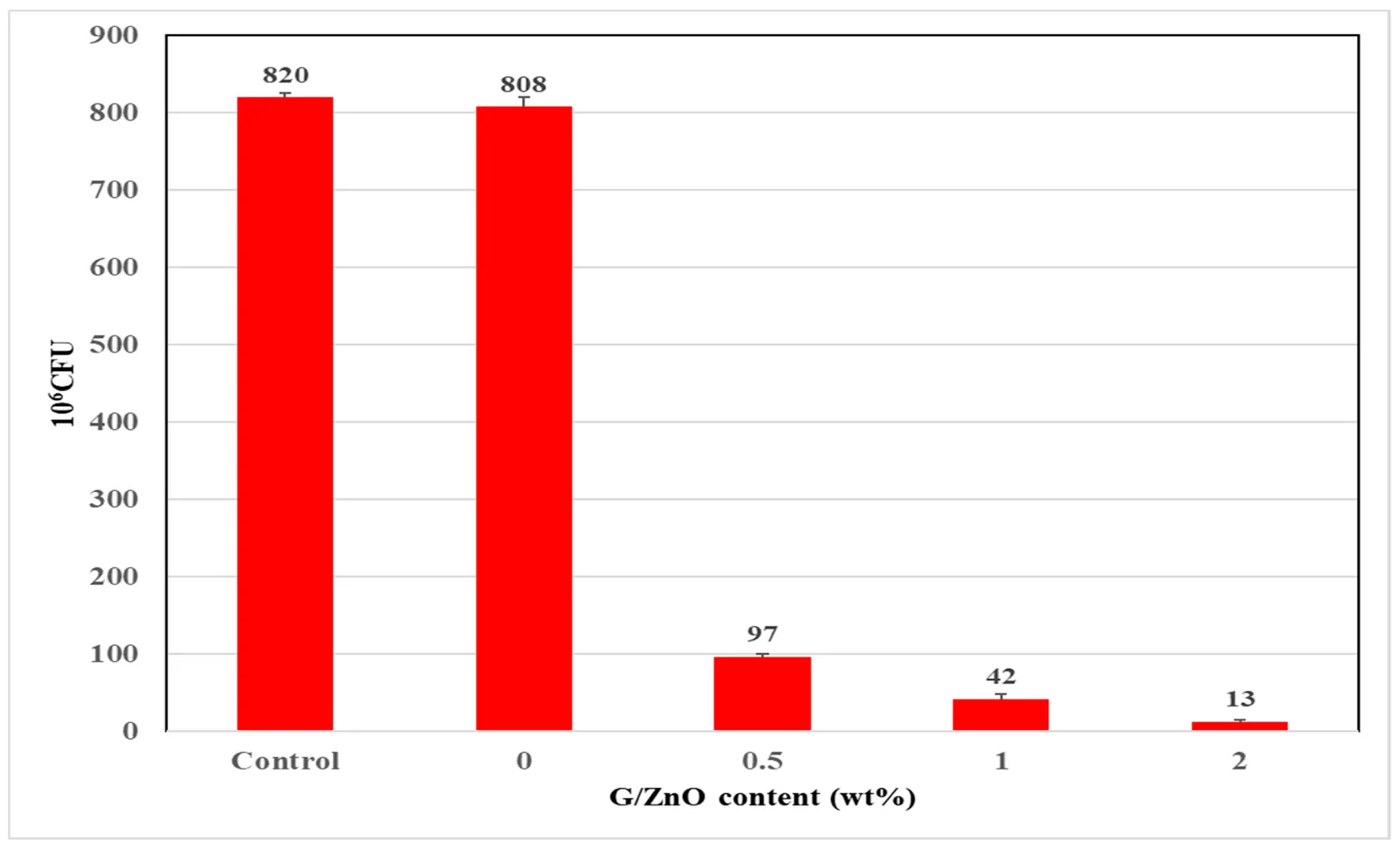
Quantitative antibacterial activity of BBD/G/ZnO/PUs against *Escherichia coli* (*E. coli*). Data are presented as the mean ± SD (*n* = 3).

**Figure 16:**
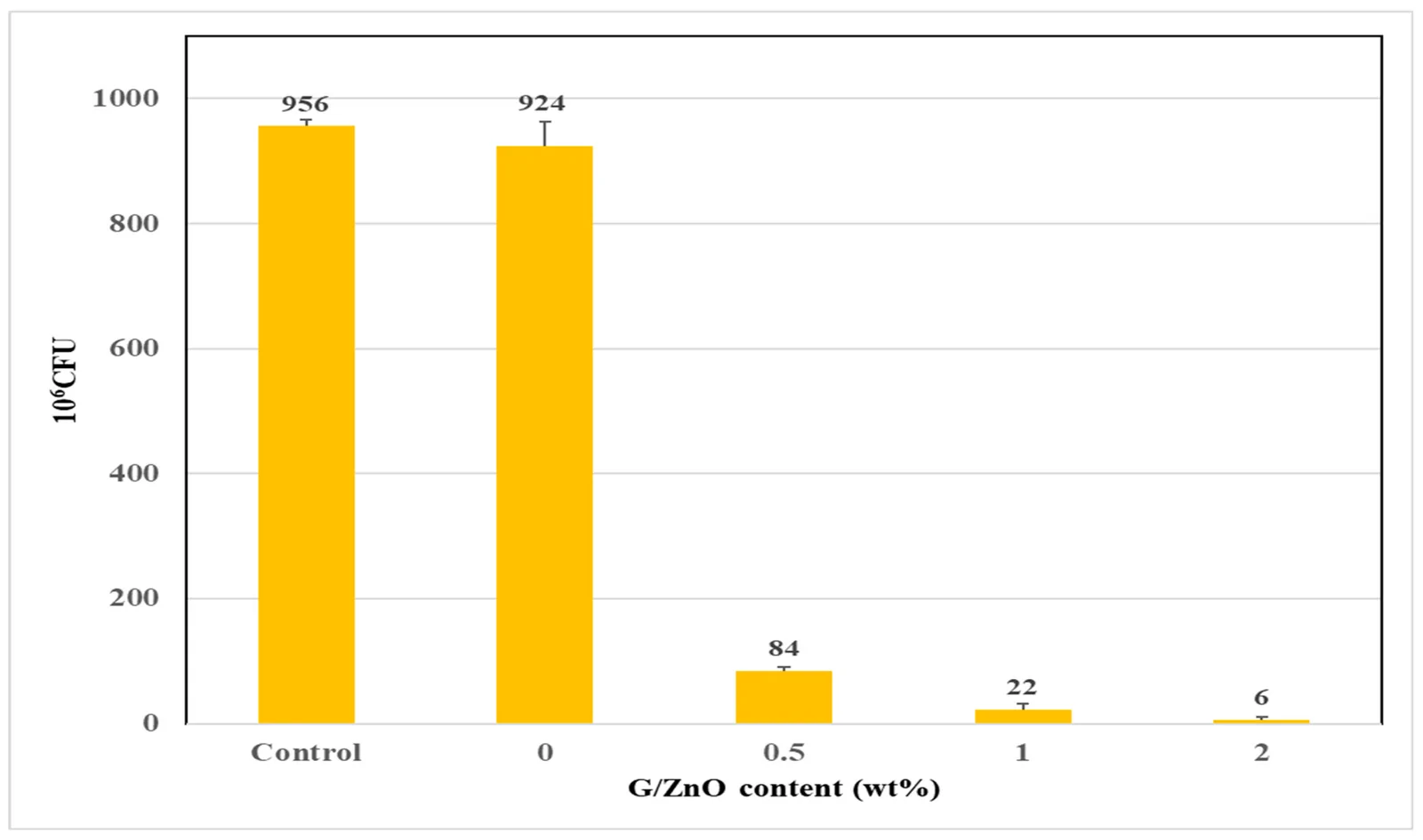
Quantitative antibacterial activity of BBD/G/ZnO/PUs against *Staphylococcus aureus* (*S. aureus*). Data are presented as the mean ± SD (*n* = 3).

The antibacterial activities of the BBD/G/ZnO/PU nanocomposites against Gram-negative *Escherichia coli* (*E. coli*) and Gram-positive *Staphylococcus aureus* (*S. aureus*) were quantitatively evaluated using the colony-forming unit (CFU) method. The antibacterial performances are presented in Figure 15 and Figure 16, and the corresponding quantitative results are summarized in Table 10 and Table 11, respectively. The neat polyurethane matrix exhibited negligible antibacterial activity, whereas the antibacterial efficiency increased progressively with increasing G/ZnO loading. The nanocomposite containing 2.0 wt.% G/ZnO (PU-04) exhibited the highest antibacterial efficiencies, reaching 98.4% against *E. coli* and 99.4% against *S. aureus*. Similar antibacterial activity has been reported for polymer systems containing graphene-, ZnO-, and graphene/ZnO-based fillers [23,24,27,28,29,38,39,40,41,42,43,46,47].

The observed antibacterial activity may be associated with the antibacterial characteristics of the components present in the commercially supplied G/ZnO hybrid filler. Previous studies have reported that graphene-based materials can interact with bacterial cell membranes through direct contact and physical disruption, whereas ZnO has been associated with antibacterial mechanisms involving reactive oxygen species (ROS), Zn^2+^ release, and interactions with bacterial cell membranes [23,24,27,38,39,40,41,42,47]. These literature-reported mechanisms may contribute to the antibacterial behavior observed in the present G/ZnO-containing composites. However, ROS generation, Zn^2+^ release, and direct membrane damage were not independently measured in the present study; therefore, a specific antibacterial mechanism cannot be confirmed from the current data.

Furthermore, because separate graphene- and ZnO-containing control samples at equivalent concentrations were not included, the individual antibacterial contributions of graphene and ZnO and any synergistic effect between these components cannot be distinguished from the present results. EDS elemental mapping confirmed only the presence and spatial distribution of Zn-containing regions within the examined areas at the resolution of SEM/EDS and does not establish the distribution of the carbonaceous/graphene component or homogeneous nanoscale dispersion of the hybrid filler. Accordingly, the observed antibacterial activity is interpreted as the overall antibacterial response associated with increasing loading of the commercially supplied G/ZnO hybrid filler within the BBD-containing polyurethane matrix.

The water contact angle increased from approximately 68° to 89° with increasing G/ZnO loading, indicating reduced apparent surface wettability. Although surface wettability can influence bacterial adhesion, bacterial adhesion behavior was not directly evaluated in the present study. Therefore, a causal relationship between the observed wettability changes and antibacterial activity cannot be established. The antibacterial efficiencies observed in the present study are generally consistent with the antibacterial behavior previously reported for graphene/ZnO-containing polymer systems [23,24,27,28,29,38,39,40,41,42,43,46,47].

Although the BBD-containing PU/G/ZnO nanocomposites exhibited changes in mechanical performance, surface wettability, and antibacterial activity under the investigated laboratory conditions, these results alone are insufficient to establish their suitability for practical coating, antibacterial-surface, or biomedical applications. Important application-related parameters, including filler leaching, long-term antibacterial stability, coating adhesion, environmental durability, and cytocompatibility, were not evaluated in the present study. These aspects require systematic investigation before practical applicability can be established.

## 4. Conclusions

A series of polyurethane (PU) nanocomposites incorporating 4,4′-bis(hydroxymethyl)-2,2′-bipyridine (BBD) as a chain extender and a commercially supplied graphene/zinc oxide (G/ZnO) hybrid filler was successfully synthesized via a two-step polymerization process. Structural characterization confirmed the formation of the polyurethane structure, while morphological observation and XRD analysis of the pristine G/ZnO hybrid filler revealed an irregular and aggregated morphology and confirmed the presence of crystalline ZnO. EDS elemental mapping showed Zn-containing regions within the examined areas of the G/ZnO-containing PU samples, while XPS analysis further confirmed the surface presence of Zn-containing species, with the Zn atomic concentration increasing with increasing G/ZnO loading. These results provide complementary evidence for the incorporation and surface presence of the Zn-containing component of the commercial G/ZnO hybrid filler but do not establish homogeneous nanoscale dispersion or a specific graphene–ZnO architecture.

Varying the G/ZnO hybrid-filler loading was associated with changes in the thermal, thermomechanical, mechanical, surface-wettability, and antibacterial properties of the BBD-containing PU system. Thermogravimetric analysis showed modest changes in thermal decomposition behavior, while DSC and DMA revealed shifts in glass-transition and relaxation behavior with increasing G/ZnO loading. The tensile strength increased from 2.68 MPa for neat PU to 11.76 MPa for the nanocomposite containing 2.0 wt.% G/ZnO, accompanied by an increase in Young’s modulus. The water contact angle increased from approximately 68° to 89°, indicating reduced apparent surface wettability with increasing G/ZnO loading. The nanocomposites also exhibited antibacterial activity against *Escherichia coli* and *Staphylococcus aureus*, with the highest antibacterial efficiencies reaching 98.4% and 99.4%, respectively, under the investigated experimental conditions.

Overall, the present results demonstrate that varying the loading of the commercially supplied G/ZnO hybrid filler modifies the structural and physicochemical properties and antibacterial behavior of the BBD-containing polyurethane system under the investigated laboratory conditions. However, because an appropriate PU system without BBD and separate graphene- and ZnO-containing control systems at equivalent concentrations were not included, the independent contribution of BBD and the respective contributions of graphene and ZnO cannot be quantitatively distinguished, and a synergistic effect between graphene and ZnO cannot be established from the present results. Therefore, the observed property changes should be interpreted as the overall effects associated with varying the loading of the commercial G/ZnO hybrid filler rather than as evidence of specific individual or synergistic contributions of its components. Furthermore, the present findings should be regarded as laboratory-scale material characterization rather than validation of practical applicability. Further studies addressing filler leaching, long-term antibacterial stability, coating adhesion, environmental durability, cytocompatibility, and other application-specific requirements are necessary before the practical applicability of these materials as functional coatings, antibacterial surfaces, or biomedical materials can be established.

## Figures and Tables

**Table 1:** Hard and soft segment contents of BBD/G/PUs.

Designation	MDI (mol)	PCL (mol)	BBD (mol)	Hard Segment (wt.%)	Soft Segment (wt.%)
BBD/G/ZnO/PUs	4	3	1	43.36	56.64

**Table 2:** Experimental formulations of BBD/G/ZnO/PUs.

Sample Name	MDI (mol)	PCL (mol)	BBD (mol)	G/ZnO (wt.%)
BBD/G/ZnO/PU-01	4	3	1	0
BBD/G/ZnO/PU-02	4	3	1	0.5
BBD/G/ZnO/PU-03	4	3	1	1
BBD/G/ZnO/PU-04	4	3	1	2

**Table 3:** Molecular-weight characteristics of BBD/G/ZnO/PU-01 determined by GPC.

Sample	Mn (×10^4^ g·mol^−1^)	Mw (×10^4^ g·mol^−1^)	PDI
BBD/G/ZnO/PU-01	2.41	4.59	1.90

**Table 4:** Elemental composition of BBD/G/ZnO/PU determined by EDS.

Sample	C (wt.%)	Zn (wt.%)
BBD/G/ZnO/PU-01	75.32	0
BBD/G/ZnO/PU-02	67.84	0.73
BBD/G/ZnO/PU-03	67.82	1.58
BBD/G/ZnO/PU-04	67.61	2.95

**Table 5:** Surface elemental compositions of the BBD/G/ZnO/PU samples determined by XPS.

Sample	C (at.%)	O (at.%)	N (at.%)	Zn (at.%)
BBD/G/ZnO/PU-01	72.42	8.93	18.65	0.00
BBD/G/ZnO/PU-02	79.39	2.23	18.15	0.23
BBD/G/ZnO/PU-03	75.20	4.22	20.29	0.29
BBD/G/ZnO/PU-04	71.86	3.86	23.46	0.82

**Table 6:** TGA data of BBD/G/ZnO/PUs.

Sample	*T*onset (°C)	*T*max (°C)	Residue at 700 °C
BBD/G/ZnO/PU-01	260.8	318.8	11.0%
BBD/G/ZnO/PU-02	262.7	325.6	11.9%
BBD/G/ZnO/PU-03	263.8	326.5	12.0%
BBD/G/ZnO/PU-04	264.8	331.3	13.3%

**Table 7:** DSC data of BBD/G/ZnO/PUs.

Sample	Tg (°C)
BBD/G/ZnO/PU-01	−13.91
BBD/G/ZnO/PU-02	−9.53
BBD/G/ZnO/PU-03	−8.15
BBD/G/ZnO/PU-04	−7.97

**Table 8:** DMA data of BBD/G/ZnO/PUs.

Sample	T_gd_ from E″ (°C)	T_gd_ from Tanδ (°C)	tan δ_max_
BBD/G/ZnO/PU-01	−7.71	−0.77	0.5218
BBD/G/ZnO/PU-02	−1.66	4.75	0.7270
BBD/G/ZnO/PU-03	1.03	7.58	0.9631
BBD/G/ZnO/PU-04	2.87	10.45	1.0258

**Table 9:** Mechanical properties of BBD/G/ZnO/PUs.

Sample	Tensile Strength (MPa)	Elongation at Break (%)	Young’s Modulus (MPa)
BBD/G/ZnO/PU-01	2.68 ± 0.69	829 ± 214	0.65 ± 0.22
BBD/G/ZnO/PU-02	3.99 ± 0.86	746 ± 160	1.24 ± 0.33
BBD/G/ZnO/PU-03	7.99 ± 1.17	708 ± 104	3.07 ± 0.51
BBD/G/ZnO/PU-04	11.76 ± 0.99	474 ± 41	5.20 ± 0.38

**Table 10:** Residual bacterial count and antibacterial efficiency of BBD/G/ZnO/PUs against *Escherichia coli* (*E. coli*).

Sample	10^6^ CFU	Antibacterial Rate (%)
Control	820	-
BBD/G/ZnO/PU-01	808	1.5
BBD/G/ZnO/PU-02	97	88.2
BBD/G/ZnO/PU-03	42	94.9
BBD/G/ZnO/PU-04	13	98.4

**Table 11:** Residual bacterial count and antibacterial efficiency of BBD/G/ZnO/PUs against *Staphylococcus aureus*.

Sample	10^6^ CFU	Antibacterial Rate (%)
Control	956	-
BBD/G/ZnO/PU-01	924	3.3
BBD/G/ZnO/PU-02	84	91.2
BBD/G/ZnO/PU-03	22	97.7
BBD/G/ZnO/PU-04	6	99.4

## Data Availability

The raw data supporting the conclusions of this article will be made available by the authors on request.
